# Quality-Gated Episode-Level RGB-D Body Condition Scoring in Beef Cattle

**DOI:** 10.3390/ani16193040

**Published:** 2026-09-27

**Authors:** Zhen Hou, Weinan Cao, Wei He, Yanwen Li, Jiarui Li, Di Wu, Ruiping Wang, Hua Yang

**Affiliations:** 1 College of Information Science and Engineering, Shanxi Agricultural University, Jinzhong 030801, China; zhenhou@sxau.edu.cn (Z.H.); caoweinan@sxau.edu.cn (W.C.); hewei2@sxau.edu.cn (W.H.); lyw@sxau.edu.cn (Y.L.); 20230302420@stu.sxau.edu.cn (J.L.); 20221210616@stu.sxau.edu.cn (D.W.); 2 Shanxi Province Science and Technology Information Research Center, Taiyuan 030027, China

**Keywords:** beef cattle, body condition scoring, RGB-D, multiple instance learning, geometric quality gating, episode-level analysis

## Abstract

In practical beef cattle monitoring scenarios, automated body condition scoring (BCS) based on single-frame visual information can be affected by posture variation, occlusion, and image quality changes. This study proposed an episode-level BCS assessment framework using synchronized RGB-D videos, integrating multi-frame visual information, attention-based multiple instance learning (Attention-MIL), and a dorsal geometry-based quality control mechanism to improve reliability under complex acquisition conditions. The publicly available Raramuri Criollo dataset was reorganized into a dataset containing 3815 RGB-D frame pairs, 52 animal identity groups, and 151 episodes, and evaluated using animal-wise three-fold cross-validation. Predictions were generated only when an episode contained at least three quality-filtered frames and reliable angle estimation. With an average coverage of 0.7345 after quality gating, the final model achieved 61.66% accuracy, a Macro-F1 score of 0.5680, and a quadratic weighted kappa (QWK) of 0.6339 for five-class BCS classification. Additionally, 89.71% of predictions were within one BCS category of the reference scores. Three-class management classification achieved a Macro-F1 score of 0.8059. Ablation experiments showed complementary contributions from RGB and depth information, and the combined angle and quality-statistic features improved several metrics in the original ablation. Additional matched experiments did not establish an independent angle benefit but supported multi-frame aggregation and within-Episode prediction consistency. The proposed RGB-D multi-frame fusion approach demonstrates potential for automated beef cattle body condition monitoring under controlled acquisition conditions.

## Simple Summary

Body condition scoring provides an important assessment of nutritional status and energy reserves in beef cattle; however, traditional manual scoring is labor-intensive and susceptible to variation among evaluators. Existing automated scoring approaches are often based on single-frame visual information, and their prediction stability may be limited under conditions involving animal posture changes, body occlusion, or reduced image quality. In this study, synchronized RGB and depth videos were used to analyze multiple frames collected from the same animal during a continuous acquisition event. A quality-gating mechanism based on dorsal geometric information was introduced, where the model did not force a prediction when an episode lacked sufficient reliable geometric information. A total of 151 episodes were analyzed. After quality gating, the final model generated predictions for approximately 73% of episodes, achieving an episode-level five-class BCS classification accuracy of 61.66%, with 89.71% of predictions within one BCS category of the reference scores. The results demonstrate that integrating multi-frame RGB-D information with dorsal morphological features has potential for automated body condition monitoring of beef cattle under controlled acquisition conditions. However, the current dataset contains a limited number of extreme body condition categories, and the model has not yet been validated in independent farm environments. Future research will focus on improving extreme-condition recognition, cross-environment generalization, and practical deployment to further advance automated cattle body condition monitoring methods.

## 1. Introduction

Body condition scoring (BCS) is an important phenotypic indicator for evaluating energy reserves, nutritional status, and production management in beef cattle, and plays a critical role in reproductive management, health monitoring, feeding regulation, and economic performance assessment [1,2]. In beef cattle production, excessively low body condition generally indicates insufficient nutrient intake or inadequate energy reserves, which may negatively affect estrus, conception, pregnancy maintenance, and calf growth. In contrast, excessively high body condition may increase feeding costs and contribute to reproductive disorders, increased locomotion burden, and metabolic risks. Therefore, establishing high-frequency, objective, and repeatable BCS monitoring methods is of great importance for improving herd management efficiency and promoting precision livestock production [3].

Traditional BCS evaluation mainly relies on trained professionals performing visual inspection and palpation. Although this approach is intuitive and relatively inexpensive, it is labor-intensive, subject to evaluator-dependent variation, and limited in assessment frequency, making it difficult to satisfy the requirements of large-scale farms for continuous, automated, and population-level monitoring [4,5]. With the rapid development of artificial intelligence technologies, computer vision and deep learning approaches have gradually matured, and image-based automated BCS assessment has become an important research direction in livestock informatics. Early approaches mainly relied on manually designed morphological features, whereas recent deep learning models, such as convolutional neural networks (CNNs), have substantially improved image representation capability and automated scoring performance [6,7,8,9,10]. Meanwhile, the application of RGB-D sensors enables simultaneous acquisition of three-dimensional structural information from the dorsal region of cattle [11,12]. Compared with conventional RGB images, depth images provide information regarding dorsal height, surface curvature, and spatial contour, which may reduce the influence of illumination conditions, coat color, and texture variations on model predictions [13,14]. However, existing RGB-D-based studies have primarily focused on incorporating depth information as part of the visual input for end-to-end feature learning. Systematic exploration remains limited regarding how to further extract biologically meaningful structural indicators from depth data and effectively integrate them with deep visual representations for complementary information fusion. Since BCS evaluation commonly considers external anatomical characteristics such as spinal prominence, skeletal visibility, and dorsal shape variation, constructing interpretable geometric features from depth information may provide additional structural cues for automated BCS assessment.

However, most existing automated BCS assessment methods have been developed under controlled environments or using manually selected high-quality images, and their stability, generalizability, and deployment reliability under practical monitoring conditions remain challenging. A common strategy in current studies is to simplify the task as single-frame image classification, where each image is treated as an independent sample for training and prediction [15]. This approach has three major limitations. First, BCS fundamentally reflects the overall fat deposition and energy reserve status of an animal rather than the visual appearance under a single instantaneous posture. Single-frame prediction is therefore vulnerable to variations in posture, body occlusion, camera viewpoint, and local image noise, limiting the utilization of complementary information contained in multiple observations within the same video sequence. Second, practical monitoring videos often contain a large proportion of unreliable frames. For example, the head, neck, or horns may enter the dorsal region, the trunk may be partially cropped, the animal may deviate from the image center, fences and background structures may introduce occlusion, and depth maps may suffer from sensor-induced missing values or noise. Such frames provide limited anatomical evaluation value, and directly feeding them into prediction models may reduce prediction stability and increase the risk of severe misclassification. Third, some video datasets exhibit strong temporal correlations. If random frame-level splitting is applied, highly similar frames collected from the same animal within a short time interval may appear simultaneously in training and testing sets, causing data leakage and inflated performance estimates [16,17]. Furthermore, in long-term monitoring datasets, the same animal may have different body condition records at different time points; therefore, experimental design must strictly control sample assignment at the individual-animal level. Existing deep learning methods largely rely on end-to-end visual feature learning, resulting in limited model interpretability. In contrast, human BCS assessment commonly considers biologically meaningful anatomical cues, including dorsal ridge prominence, skeletal visibility, and body surface smoothness. Effectively integrating such structural features with deep visual representations represents an important direction for improving model reliability and interpretability [18,19].

Based on the above challenges, this study aims to develop an automated BCS assessment framework designed for practical beef cattle monitoring scenarios. The proposed framework integrates multi-frame information from RGB-D videos, actively excludes low-quality observations, and incorporates biologically meaningful dorsal morphological features to improve the reliability and applicability of automated body condition assessment. The main contributions of this study are summarized as follows:

1. Development of an episode-level BCS assessment paradigm for beef cattle. Unlike conventional single-frame classification approaches, this study treats RGB-D video segments collected from the same animal during a specific body condition period as an Episode and models each Episode as a sample bag containing multiple frame-level instances. Attention-based Multiple Instance Learning (Attention-MIL) is employed to aggregate multi-frame information, enabling comprehensive assessment of body condition status and better reflecting the biological characteristics of BCS as an animal-level attribute. It should be noted that episode-level modeling does not simply increase the number of repeated observations from the same animal; rather, it exploits complementary information from different postures and quality levels during continuous acquisition and reduces the influence of abnormal single-frame observations through multi-instance aggregation.
2. Introduction of a geometric quality-gating strategy. This study performs geometric quality assessment for frames within each Episode, automatically identifying and excluding unreliable observations that do not meet predefined quality criteria. Only frames passing geometric quality control are used for model training and prediction. When an Episode contains insufficient valid frames or fails to provide reliable dorsal angle features, the system does not force a prediction output, thereby reducing the risk of high-confidence errors caused by low-quality inputs.
3. Fusion of biologically meaningful dorsal ridge angle features. This study extracts the Episode-level median dorsal ridge angle from depth images that pass quality control and calculates the Good-frame ratio and Good-angle-frame number as reliability statistics for angle estimation. These three features are preprocessed and fused with RGB-D visual representations at the feature level, providing biologically meaningful geometric information to complement end-to-end visual feature learning.

## 2. Materials and Methods

This study focuses on RGB-D multi-frame data-based BCS in beef cattle and develops a comprehensive assessment framework by integrating multiple instance learning and geometric quality gating. Figure 1 illustrates the overall workflow of the system from data input to prediction output. First, RGB images and depth maps are imported and paired, followed by basic preprocessing and the construction of an episode-level data index. Subsequently, animal identities are normalized, and animal-wise three-fold cross-validation is applied. The geometric quality-gating module then evaluates each frame based on criteria including trunk-region completeness, posture suitability, dorsal contour continuity, and the availability of computable cross-sectional angle information. For the quality-gated model, instances are sampled only from frames passing quality control. Parallel CNNs are employed to extract RGB and depth visual features, which are integrated through frame-level fusion and aggregated using Attention-MIL to generate episode-level visual representations. Meanwhile, the episode-level dorsal ridge angle and its reliability-related features are encoded as geometric representations and fused with the visual representations. Finally, the model outputs episode-level five-class BCS predictions, which are further converted into three-level body condition categories according to predefined mapping rules. Samples that do not satisfy episode-level quality criteria are excluded from the final prediction set.

### 2.1. Data and Classify

#### 2.1.1. Data Source and Label Description

This study used the publicly available dataset “Labeled RGB and Depth Images for Cattle Body Condition Score Prediction” [20]. This dataset contains data collected from a Raramuri Criollo cattle herd in New Mexico, USA. During data acquisition, approximately one-third of the cattle were allowed to remain standing inside the chute, while the remaining animals were guided by personnel through the imaging area along the handling chute. Synchronized RGB and depth videos were collected using an Intel RealSense D435i RGB-D camera (Intel Corporation, Santa Clara, CA, USA) mounted above the chute. The camera recorded RGB videos and depth videos at a resolution of 640 × 480 pixels (with a small number of recordings at 848 × 480 pixels, which were unified during preprocessing), and the data were stored in the Robot Operating System (ROS) bag format. BCS labels were provided by the original dataset and followed the commonly used 1–9 integer BCS scoring system in the United States. These scores were obtained by professional veterinarians through visual assessment combined with palpation. The original ROS bag files stored RGB frames and depth maps indexed by timestamps and contained continuous observations of the same animals collected at different acquisition time points. In this study, synchronized RGB images and depth maps were first extracted frame-by-frame from each ROS bag according to timestamps. After complete parsing, a total of 5940 synchronized RGB-D frame pairs were obtained. Before model development, manual data inspection was conducted to remove frames that could not provide valid animal observations. During this pre-modeling screening process, 2125 frame pairs were excluded, leaving 3815 synchronized RGB-D frame pairs for subsequent analysis. It should be noted that this preliminary cleaning step only addressed data availability issues and did not involve sample selection based on BCS category, animal posture, dorsal morphology, or model prediction difficulty. Subsequent filtering related to posture, occlusion, dorsal contour quality, and depth-based geometric reliability was performed by the quality-gating module proposed in this study.

In this dataset, BCS labels covered five levels from BCS 2 to BCS 6. Samples with BCS 4 and BCS 5 accounted for a relatively large proportion, resulting in apparent class imbalance. Depth maps were spatially aligned with RGB images, providing corresponding depth information for each pixel. It should be noted that this study directly adopted the BCS labels provided by the public dataset and did not reorganize an independent scoring process; therefore, inter-observer agreement metrics (e.g., Fleiss’ kappa) could not be recalculated using the current study data. The existing labels were generated according to the standardized BCS scoring procedure in the original dataset and were used as reference annotations for the present study.

#### 2.1.2. Episode Indexing

Unlike conventional classification tasks that treat individual images as independent samples, this study defined a group of synchronized RGB-D observations collected from the same animal during one continuous acquisition event as an Episode and considered it as a sample bag in Multiple Instance Learning (MIL) [18,19]. Each Episode contains multiple synchronized RGB-D frame instances, and the model performs Episode-level BCS prediction by aggregating information from multiple instances. It should be noted that an Episode represents a continuous acquisition event rather than a single frame image. The same physical animal may generate multiple Episodes at different acquisition time points. Therefore, Episode-level prediction avoids treating consecutive observations from the same animal as completely independent samples and better reflects the biological characteristics of BCS as an animal-level body condition indicator.

#### 2.1.3. Animal Identity Normalization in Longitudinal Data

Due to the longitudinal characteristics of the dataset, the same physical animal may have body condition records collected at different time periods. For example, records named as Cow_5, Cow_5_1, or Cow_5_2 represent acquisition results from the same animal at different body condition stages. After identity normalization, the 151 Episodes were assigned to 52 Animal identity groups. Following the above data organization procedure, 3815 valid synchronized RGB-D frame pairs were ultimately organized into 151 Episodes, corresponding to 52 Animal identity groups, as summarized in Table 1.

#### 2.1.4. Five-Class BCS Task and Three-Class Management Classification

Each Episode was assigned a corresponding BCS label. The original task was formulated as a five-class body condition classification problem, including five categories from BCS 2 to BCS 6. To better reflect practical decision-making requirements in cattle management, the five-class BCS labels were further mapped into three management categories. Specifically, BCS 2 and BCS 3 were grouped as Lean, representing low body condition or thin animals; BCS 4 and BCS 5 were grouped as Ideal, representing appropriate body condition; and BCS 6 was categorized as High, representing excessive body condition.

### 2.2. Cross-Validation and Geometric Quality Control

#### 2.2.1. Animal-Wise Three-Fold Cross-Validation

This study employed animal-wise three-fold cross-validation [16,17]. The three folds contained 51, 50, and 50 Episodes, corresponding to 18, 17, and 17 Animal identity group identifiers, respectively. In each experimental round, one fold was used as an independent test set, while the remaining two folds were used as the training and validation candidate set. Subsequently, approximately 22% of animals from the non-test animals were randomly selected as the validation set, and the remaining animals were used for training. This proportion was determined according to the number of available animals to maintain a reasonable dataset size among the training, validation, and test sets. The validation split was not stratified by BCS category and did not enforce preservation of rare classes. Therefore, due to the limited sample size, differences in class composition among folds may exist. This study prioritized animal-level separation to prevent Episodes from the same Animal identity group appearing across the training, validation, and test sets. To ensure input consistency, a frame-level index table was first constructed for the RGB images and depth maps. The index table recorded the path of each RGB image, the path of the corresponding depth map, the episode identifier, the BCS label, the three-category body condition label, and the corresponding animal identifier. Each episode consisted of a sequence of frames collected from the same animal during the same body condition period. Therefore, frames within the same episode exhibited strong temporal correlation and visual similarity. During subsequent modeling, individual frames were no longer treated as independent prediction samples but instead served as instances within an episode for multiple-instance learning.

#### 2.2.2. Frame-Level Quality Criteria

This study adopted a quality control strategy based on RGB-D geometric information rather than explicitly detecting semantic objects such as heads, horns, fences, or other interfering structures. The system evaluated each frame according to the completeness of the candidate trunk region, animal centering, posture suitability, dorsal contour continuity, and the availability of computable cross-sectional angles. Cases involving head or neck intrusion, horn occlusion, trunk truncation, severe inclination, background interference, or missing depth information were identified as low-quality frames by triggering corresponding geometric quality failure conditions. A frame was defined as a Good-frame only when it simultaneously satisfied all five criteria: completeness, centering, posture suitability, contour continuity, and angle computability.

#### 2.2.3. QC-Based Dorsal Ridge Angle Extraction

Dorsal ridge prominence and contour smoothness are important visual cues in manual BCS assessment. Animals with lower body condition generally exhibit sharper and more prominent dorsal contours, whereas animals with higher body condition tend to show relatively broader and smoother dorsal profiles. Therefore, this study extracted dorsal ridge angles from Depth Maps that passed quality control and used them as auxiliary geometric features. It should be noted that the angle was calculated within a two-dimensional cross-sectional contour coordinate system after frame-level depth normalization and posture correction, rather than being an absolute three-dimensional geometric angle derived from calibrated point clouds. The algorithm applied principal component analysis to correct trunk inclination within the image plane and identified a relatively stable trunk region based on the width profile of the animal body, thereby reducing the effects of lateral displacement, scale variation, and trunk rotation. However, camera perspective and three-dimensional posture variations may still introduce certain influences on the estimated angle. For each candidate cross-section, the depth contour was first smoothed, and the maximum value of the smoothed contour was operationally defined as the dorsal ridge vertex. This vertex was required to be located within the central 35–65% region of the body width. Subsequently, contour points within 5–40% of the relative body width range from the vertex on both sides were selected for first-order ordinary least squares linear fitting. Each side required at least four valid points, and the angle between the two fitted lines was calculated based on their directional vectors. The current method did not apply an independent point-level outlier removal procedure or a goodness-of-fit threshold. Instead, unreliable cross-sections were excluded through quality conditions including contour smoothing, peak position, peak number, peak prominence, trunk completeness, and centerline stability. A smaller angle indicates a relatively sharper normalized dorsal contour, whereas a larger angle indicates a relatively broader and flatter contour. This feature reflects external dorsal morphological characteristics associated with body condition variation rather than directly measuring fat thickness or fat deposition. Figure 2 illustrates the dorsal ridge angle extraction procedure.

#### 2.2.4. Definition of Good-Angle Frame and Episode Eligibility

Based on Good-frame selection, an Episode was included in the quality-gated prediction set only when it contained at least three Good-angle frames and a non-missing Episode-level median dorsal ridge angle could be calculated. In this study, a frame was defined as a Good-angle frame when it did not trigger any frame-level quality failure conditions, contained no more than nine candidate cross-sections, and provided at least three valid cross-sectional angle measurements, as illustrated in Figure 3.

It should be noted that frames producing one or more cross-sectional angles but failing to satisfy all quality control criteria were not counted as Good-angle frames. Among them, $n_{good}^{\left(i\right)}$ represents the number of Good-angle frames in an Episode, and $\tau_{frame}$ was defined as the minimum frame threshold.

(1) $$Coverage=\frac{1}{\left|D_{test}\right|}\sum_{B_{i}\in D_{test}}I\left[Eligible\left(B_{i}\right)\right],$$

(2) $$Eligible\left(B_{i}\right)=I\left(n_{good}^{\left(i\right)}\geq \tau_{frame}\right)\cap I\left(\phi_{median}^{\left(i\right)}\neq NaN\right).$$

For models incorporating quality gating, this study reported the Coverage metric. Coverage was defined as the proportion of Episodes satisfying quality-gating criteria and receiving predictions relative to the total number of Episodes in the test set. This metric reflects the effective prediction range after rejecting low-quality inputs. In practical deployment scenarios, there is a trade-off between Coverage and prediction reliability. Higher Coverage indicates that the system can provide predictions for more samples, whereas stricter quality gating may reduce Coverage but help reduce high-risk errors caused by unreliable inputs.

To avoid pseudo-replication caused by multiple observations within the same Episode, frame-level angle measurements were aggregated into Episode-level features. Each Good-angle frame could generate multiple valid cross-sectional angle measurements. For Episode $B_{i}$, all valid cross-sectional angles generated from its Good-angle frames were combined, and the median of the combined angle set, denoted as $\phi_{median}^{\left(i\right)}$, was used as the Episode-level representative angle. This aggregation strategy reduces the influence of individual abnormal cross-sections or occasional abnormal frames on angle estimation.

In addition to the median dorsal ridge angle, two statistical features reflecting the reliability of angle estimation were calculated. Let $N_{all}^{\left(i\right)}$ denote the total number of available frames processed during quality control, and $n_{good}^{\left(i\right)}$ denote the number of Good-angle frames among them. The Good-frame ratio was defined as:

(3) $$r_{good}^{\left(i\right)}=\frac{n_{good}^{\left(i\right)}}{N_{all}^{\left(i\right)}}.$$

Before being input into the classification model, the number of Good-angle frames was transformed using $log\left(1+n_{good}^{\left(i\right)}\right)$ to reduce the influence of different Episode lengths on feature scale. The final three Episode-level angle-related features were the median dorsal ridge angle, Good-frame ratio, and transformed Good-angle-frame number.

### 2.3. Multimodal Model Training

#### 2.3.1. Parallel RGB-D Encoding and Episode-Level Visual Representation

Each episode was defined as a multiple-instance learning bag, with an individual video frame serving as an instance. For full-coverage model variants without quality gating, instances were sampled from all original RGB-D frames in the episode. For quality-gated models, instances were sampled only from valid frames that passed geometry-based quality control, namely Good frames.

To standardize the input length across episodes, 12 frames were sampled from each episode. During training, when fewer than 12 frames were available, random sampling with replacement was used. During validation and testing, frames were first sampled at approximately equal intervals. When fewer than 12 valid frames were available, the final frame was repeated until the required input length was reached.

For each sampled frame, the RGB image and depth map were independently fed into two lightweight CNN encoders with separate parameters. The RGB encoder received a three-channel RGB image, whereas the depth encoder received a single-channel depth map. The two encoders had the same architecture and each consisted of four convolutional stages with channel dimensions of 32, 64, 128, and 192. Batch normalization, ReLU activation, max pooling, and adaptive global average pooling were applied, and each encoder produced a 128-dimensional modality-specific feature [21].

For the t-th frame, the RGB and depth features were denoted as $f_{t}^{rgb}\in R^{128}$ and $f_{t}^{depth}\in R^{128}$ respectively. The two modality-specific features were concatenated along the channel dimension: $f_{t}=[f_{t}^{rgb};f_{t}^{depth}]$, resulting in a 256-dimensional frame-level RGB-D joint feature. This joint feature was further processed through a linear projection layer, ReLU activation, and dropout with a rate of 0.20 to obtain the frame-level multimodal representation $h_{t}\in R^{256}$. The set of frame-level representations within an episode, $H=\left\{h_{1},h_{2},\ldots ,h_{T}\right\}$ was then provided to the Attention-MIL module. Attention-MIL learned instance weights according to the contribution of each frame to episode-level body condition assessment [19]:

(4) $$\alpha_{t}=\frac{exp(a(h_{t}))}{\sum_{j=1}^{T}exp(a(h_{j}))},$$

where $\alpha_{t}$ denotes the attention weight assigned to the t-th frame. The final episode-level visual representation was obtained through weighted aggregation:

(5) $$z_{v}=\sum_{t=1}^{T}\alpha_{t}h_{t},$$

where $z_{v}$ denotes the episode-level visual representation. This episode-level modeling strategy avoided treating individual frames as independent prediction units and enabled the model to integrate information from multiple views and postures captured from the same animal over a short period. This formulation better reflects the biological meaning of BCS as an overall body condition attribute rather than a characteristic of an instantaneous posture.

#### 2.3.2. Geometric Feature Encoding and Visual–Multimodal Fusion

In addition to RGB-D visual information, biologically meaningful episode-level geometric features were introduced to improve the model’s sensitivity to body condition-related morphological changes.

For each episode, a three-dimensional geometric feature vector was constructed:

(6) $$x_{a}=[\phi_{median}^{\left(i\right)},r_{good}^{\left(i\right)},\log(1+n_{good}^{\left(i\right)})],$$

where $\phi_{median}^{\left(i\right)}$ denotes the median calculated after pooling all valid transverse-section angles obtained from all Good-angle frames within the episode; $r_{good}^{\left(i\right)}$ denotes the proportion of Good-angle frames among all available frames processed during the quality-control stage; and $n_{good}^{\left(i\right)}$ denotes the number of Good-angle frames that triggered no frame-level quality-failure condition and yielded at least three valid transverse-section angles. It should be noted that these reliability features were calculated from all available frames processed during quality control and were independent of the fixed sampling of 12 frames subsequently used by the Attention-MIL visual branch.

These features were standardized independently within each cross-validation fold. Specifically, the mean and standard deviation were calculated using only the training set, and the same parameters were subsequently applied to the validation and test sets to avoid data leakage. The standardized three-dimensional geometric feature vector was input into a lightweight fully connected encoder: $z_{a}=\mathrm{MLP}(x_{a})$, where the MLP consisted of two fully connected layers with ReLU activation and dropout with a rate of 0.10. The encoder produced a 32-dimensional geometric representation: $z_{a}\in R^{32}$. The episode-level visual representation and geometric representation were then concatenated: $z_{f}=[z_{v};z_{a}]$, where $z_{v}$ was the 256-dimensional visual representation and $z_{a}$ was the 32-dimensional geometric representation. The resulting 288-dimensional joint feature was passed through a shared projection layer: $z_{s}=Wz_{f}+b$, producing a 192-dimensional shared fused representation: $z_{s}\in R^{192}$. This shared fused representation was provided simultaneously to the main five-class BCS classification head and two auxiliary classification heads. The main head generated the final BCS 2–6 prediction, whereas the Low-condition and High-condition auxiliary branches provided additional supervision only during training and were not used during final inference.

#### 2.3.3. Main Classification Head and Auxiliary Heads

The main prediction task was five-class BCS classification. The shared fused representation was provided to the five-class BCS head, which generated five logits corresponding to BCS 2–6. During inference, the logits were converted to class probabilities using the softmax function, and the class with the highest probability was selected as the episode-level BCS prediction. The final three-category body condition classification was obtained by deterministically mapping the five-class BCS prediction to Lean, Ideal, or High. The Low-condition and High-condition auxiliary heads were not used during inference.

Because animals with extreme body conditions have high warning value in herd management, Low-condition and High-condition auxiliary branches were introduced during training. The Low-condition branch received the shared fused representation and performed a binary classification task to determine whether the current episode belonged to the Lean category. Specifically, BCS 2 and BCS 3 were defined as Lean, whereas BCS 4, BCS 5, and BCS 6 were defined as non-Lean. The Low-condition auxiliary branch generated a binary logit indicating whether the current episode belonged to the Lean category. During training, BCS 2 and BCS 3 were assigned positive labels, whereas BCS 4, BCS 5, and BCS 6 were assigned negative labels, and the branch was optimized using binary cross-entropy loss.

Similarly, the High-condition auxiliary branch received the shared fused representation and performed a High-versus-non-High binary classification task. BCS 6 was defined as High, whereas BCS 2, BCS 3, BCS 4, and BCS 5 were defined as non-High.

#### 2.3.4. Training Settings and Loss Functions

The models were trained under the animal-wise three-fold cross-validation framework. In each round, one fold was used as the test set, whereas the remaining two folds formed the candidate pool for training and validation. Approximately 22% of the candidate animals were randomly selected for validation. Each model was trained for 70 epochs. Input images were resized to 160 × 160 pixels, each episode contained 12 input frames, the batch size was 8, and the number of DataLoader workers was 4. AdamW was used as the optimizer [22], with an initial learning rate of 2 × 10^−4^ and a weight-decay coefficient of 1 × 10^−4^. A cosine annealing learning-rate schedule was applied [23]. Automatic mixed precision and gradient clipping with a maximum norm of 5.0 were enabled during training.

RGB images were resized to 160 × 160 pixels and normalized to approximately the range [−1, 1]. Each depth map was first center-cropped using a crop ratio of 0.70. The 1st and 99th percentiles were then calculated using only valid nonzero pixels, followed by percentile clipping and normalization. The depth values were subsequently inverted into a closeness representation so that closer or higher regions had larger values. Finally, the depth maps were resized to 160 × 160 pixels.

Model parameters were learned by minimizing the expected total loss under the effective training sampling distribution $q_{train}$, which was induced by inverse-class-frequency weighted sampling:

(7) $$\theta_{opt}=\arg\underset{\theta }{\min}E_{\left(B_{i},y_{i}\right)∼q_{train}}\left[L_{total}\left(f_{\theta }\left(B_{i}\right),y_{i}\right)\right].$$

Model parameters were optimized using AdamW, which is written compactly as:

(8) $$\theta_{t+1}=\mathrm{AdamWUpdate}\left(\theta_{t},{𝛻}_{\theta }L_{total};\eta_{t},\lambda_{wd}\right),$$

where $\eta_{t}$ denotes the learning rate at training step t under cosine annealing, and $\lambda_{wd}$ denotes the weight-decay coefficient.

The composite checkpoint-selection score on the validation set was defined as:

(9) $$S_{t}={\mathrm{MacroF}1}_{3}\left(D_{val};\theta_{t}\right)+0.35{\mathrm{QWK}}_{5}\left(D_{val};\theta_{t}\right)-0.04P_{extreme,t},$$

(10) $$P_{extreme,t}=2E_{BCS2\to BCS6,t}+E_{BCS3\to BCS6,t}+2E_{BCS6\to BCS2/3,t}.$$

Here, $P_{extreme,t}$ denotes the weighted extreme-error penalty at epoch t. To alleviate class imbalance in the five-class BCS task, a WeightedRandomSampler with sampling weights inversely proportional to class frequency was applied to the training set, and inverse-frequency class weights were also used in the five-class cross-entropy loss. Image-level augmentation methods such as random horizontal flipping and small-angle rotation were not used [24].

All models were trained for the full 70 epochs without terminating training early. The checkpoint with the highest composite validation score was retained:

(11) $$t^{*}=\arg\underset{1\leq t\leq 70}{\max}S_{t},\hat{\theta }=\theta_{t^{*}}.$$

Here, $t^{*}$ denotes the epoch with the highest composite validation score, and $\hat{\theta }$ denotes the model parameters corresponding to that epoch and ultimately used for test-set evaluation.

For models containing the Low-condition and High-condition auxiliary branches, the total training loss consisted of the main five-class BCS classification loss $L_{BCS}$, the Low-condition auxiliary loss $L_{low}$, and the High-condition auxiliary loss $L_{high}$:

(12) $$L_{total}=L_{BCS}+0.45L_{low}+0.45L_{high}.$$

For the RGB-depth CO-MIL model without Low-condition and High-condition auxiliary supervision, the total loss consisted only of $L_{BCS}$. The main five-class BCS task used class-weighted cross-entropy loss, whereas the Low-condition and High-condition auxiliary tasks each used binary cross-entropy loss.

### 2.4. Ablation Study Design and Evaluation Metrics

#### 2.4.1. Ablation Study Design

To evaluate the contributions of input modalities, Low-condition and High-condition auxiliary supervision, geometric quality gating, and Episode-level angle-related features, seven model variants were constructed, as summarized in Table 2. Unless otherwise specified, all variants used the same animal-wise three-fold cross-validation splits, training settings, number of sampled frames, and evaluation procedures.

For Models 1–4, instances were sampled from all available RGB-D frames within each Episode, and predictions were generated for the complete test set. In Model 5, instances were sampled only from quality-filtered frames. An Episode was considered eligible for prediction only when it contained at least three Good frames and produced a non-missing Episode-level median angle. Although the angle estimation was used to define the eligible Episode set, Model 5 did not provide any angle-related features to the classification network. Model 6 adopted the same quality-gating criteria and the same eligible Episode set as Model 5, but additionally incorporated the Episode-level median dorsal ridge angle, Good-frame ratio, and valid angle frame number. Therefore, the comparison between Models 5 and 6 under matched conditions was used to evaluate the incremental contribution of angle-related features. The Angle-only MLP model used only three Episode-level angle-related features without RGB, depth information, or the Attention-MIL visual branch. It consisted of a two-layer fully connected network with a hidden dimension of 64 and retained the main five-class BCS classification head as well as the Low-condition and High-condition auxiliary training heads. The comparison among RGB-only, Depth-only, and RGB-depth LowHigh CO-MIL models was used to evaluate the individual and complementary contributions of RGB appearance information and depth-based structural information. The comparison between RGB-depth CO-MIL and RGB-depth LowHigh CO-MIL was used to assess the effect of auxiliary supervision. Because quality-gated and non-gated models were evaluated on different Episode sets, their performance should be interpreted together with Coverage rather than considered as a strict single-factor comparison. The comparison between Models 5 and 6 quantified the contribution of angle-related features under identical eligibility conditions, whereas the Angle-only MLP model examined whether these low-dimensional geometric features alone were sufficient for BCS prediction without visual representations.

The comparison of Models 5 and 6 tests the angle and both quality statistics jointly, not the angle alone. Likewise, “Angle-only MLP” denotes a three-feature geometric model. Their separate contributions were examined in the additional matched analysis below.

To control for evaluation-set differences between gated and non-gated models, an additional matched-subset analysis was performed. The RGB-depth LowHigh CO-MIL model was re-evaluated using only the dataset that passed quality gating. This evaluation condition was identical to that used for the Quality-gated RGB-depth LowHigh, no angle model. Therefore, the two models were compared using the same dataset and the same three-fold cross-validation scheme.

#### 2.4.2. Evaluation Metrics

Model performance was evaluated from four aspects: five-class BCS prediction, three-class management classification, quality-gating Coverage, and extreme misclassification errors. For the five-class BCS classification task, the following metrics were used: overall accuracy (Acc5), Macro-F1, Weighted-F1, quadratic weighted kappa (QWK5) [25], mean absolute error (MAE), and Within-one accuracy. Within-one accuracy represents the proportion of samples for which the difference between predicted BCS and reference BCS was within one category. Since BCS is an ordinal variable, errors between adjacent categories are generally more acceptable in practical management than errors spanning multiple categories. Therefore, Within-one accuracy is an important metric for evaluating the practical utility of BCS prediction models. The three-class management classification results were obtained by mapping the five-class BCS predictions into management categories. The evaluation metrics included Acc3, Macro-F1, QWK3, Lean recall, and High recall. Compared with overall accuracy, Macro-F1 and category-specific recall provide better insight into the model’s ability to recognize low- and high-condition animals under class imbalance.

All dorsal ridge angle-related analyses were performed at the Episode level to avoid pseudo-replication caused by multiple observations within the same Episode. Spearman rank correlation analysis was used to evaluate the monotonic relationship between the Episode-level median dorsal ridge angle and the five-class BCS labels. The Kruskal–Wallis H test was applied to compare differences in angle distributions among the five BCS categories [26]. For the three management categories (Lean, Ideal, and High), pairwise comparisons were performed using two-sided Mann–Whitney U tests. Multiple comparison *p*-values were corrected using the Benjamini–Hochberg method [27], and Cliff’s δ was reported as the effect size [28]. Model performance was first calculated independently on each test fold and then summarized as the mean and standard deviation across the three folds. Confusion matrices and extreme error counts were calculated after merging out-of-fold test predictions from all three folds. Ablation experiments primarily compared metric changes among model variants under the same Animal-wise split scheme. For models with different Coverage values, results were interpreted jointly with prediction Coverage.

#### 2.4.3. Additional Component and Prediction Assessment

The additional experiments evaluated 111 quality-eligible Episodes from 52 animals, with 42, 36 and 33 test Episodes across the three folds and training seeds 2026, 2027 and 2028. Four matched variants used RGB-D attention pooling with no varying geometric features (V), angle only (VA), the Good-frame ratio and log-transformed Good-angle-frame count only (VQ), or all three features (VAQ). The geometric encoder was retained in every variant; excluded coordinates were zeroed after training-only standardization to control model capacity. Eligibility, animal splits and auxiliary supervision were held fixed across variants. Single-frame and 12-frame mean-pooling visual controls assessed aggregation. These were separate retraining experiments, not replacements for the original runs. Metrics were computed from pooled out-of-fold predictions for each seed and summarized as mean ± SD across seeds; Macro-F1 used fixed class sets. The primary contrast was VAQ minus VQ in Acc5. Paired 95% percentile intervals used 2000 animal-cluster bootstrap samples within test folds, preserving model and seed pairing and averaging seed-specific metrics. Intervals are conditional on fitted models; secondary comparisons are exploratory.

Prediction consistency was evaluated using repeated samples of 1, 3, 6 and 12 quality-filtered frames from held-out Episodes with frozen models. Sampling used replacement only when too few frames were available. Full-Episode geometric features were held fixed in this visual-sampling test. A separate analysis split indexed frames into two non-overlapping halves in frame-key order, recomputed geometric features and eligibility within each half, and withheld predictions for ineligible halves. Agreement was evaluated alongside reference-label accuracy and both-half eligibility. These tests assess within-acquisition prediction consistency, not observer agreement or external validity.

## 3. Results

### 3.1. Dataset Distribution and Quality-Control Analysis

The final dataset constructed in this study consisted of 151 Episodes and 3815 valid synchronized RGB-D frame pairs, which were divided according to 52 Animal identity groups using an animal-wise split strategy. As shown in Table 3 and Figure 4, the Episode-level BCS distribution included 6 Episodes with BCS 2, 20 Episodes with BCS 3, 50 Episodes with BCS 4, 51 Episodes with BCS 5, and 24 Episodes with BCS 6, corresponding to proportions of 3.97%, 13.25%, 33.11%, 33.77%, and 15.89%, respectively. Among these, BCS 4 and BCS 5 together accounted for 66.89% of all Episodes, whereas BCS 2 accounted for only 3.97%, indicating a substantial class imbalance in the Episode-level labels.

As shown in Table 4 and Figure 5, approximately 48.9% of all frames (1865 frames) passed the geometric quality control criteria. The frame-level distribution was not identical to the Episode-level distribution. In this study, Episode was used as the fundamental evaluation unit, whereas frame-level statistics were reported only for quality analysis.

Among the 3815 frame pairs, BCS 2, 3, 4, 5, and 6 contained 758, 332, 883, 682, and 1160 frame pairs, respectively, accounting for 19.9%, 8.6%, 23.1%, 17.9%, and 30.5% of all frames. Therefore, this study reported both Episode-level and frame-level statistics when describing class distributions, while model training and evaluation were performed using Episode as the basic sample unit.

### 3.2. Biological Relevance of the Dorsal Ridge Angle

As shown in Table 5 and Figure 6, among the 151 Episodes, 8 Episodes were excluded due to insufficient valid angle measurements, and Episode-level dorsal ridge angles were successfully calculated for the remaining 143 Episodes. The Episode-level dorsal ridge angle showed a moderate positive correlation with BCS scores (Spearman’s ρ = 0.440, *p* < 0.001). The Kruskal–Wallis test indicated significant differences in angle distributions among different BCS categories (H = 39.107, *p* < 0.001). Further pairwise comparisons using the Mann–Whitney U test with Benjamini–Hochberg false discovery rate correction showed that all pairwise comparisons among the Lean, Ideal, and High groups reached statistical significance (adjusted *p* < 0.01). Effect size analysis indicated a moderate effect between Lean and Ideal (Cliff’s δ = −0.355), while Lean–High and Ideal–High comparisons showed large effects (δ = −0.815 and −0.620, respectively).

A negative Cliff’s δ value indicates that the angle distribution of the former group is generally lower than that of the latter group. These results indicate that the dorsal ridge angle generally increased with increasing body condition category, with the largest difference observed between the Lean and High groups. However, considering the distributional overlap among different categories, this angle is more appropriately used as complementary information to RGB-D visual features rather than as an independent feature for BCS classification.

### 3.3. Performance of the Final Model

The Final quality-gated angle-aware CO-MIL model achieved an average three-fold Coverage of 0.7345 ± 0.0827 on the test Episodes that satisfied the quality-gating criteria, as shown in Table 6.

As shown in Table 7, for the five-class BCS classification task, the model achieved an Acc5 of 0.6166 ± 0.1208, a Macro-F1 of 0.5680 ± 0.1504, a Weighted-F1 of 0.5952 ± 0.1430, a QWK of 0.6339 ± 0.2865, an MAE of 0.5166 ± 0.2397, and a Within-one accuracy of 0.8971 ± 0.0688.

After mapping the five-class predictions into three management categories (Lean, Ideal, and High), the model achieved an accuracy of 0.8367 ± 0.0506, a Macro-F1 of 0.8059 ± 0.0816, and a QWK of 0.7603 ± 0.1300. The recalls for the Lean and High categories were 0.6667 ± 0.1667 and 1.0000, respectively.

A High recall of 1 indicates that, among the BCS 6 Episodes included in prediction after quality gating across the three test folds, no Episode was misclassified as a non-High category. The average Coverage was 0.7345, meaning that approximately 26.5% of the original test Episodes were excluded from prediction by the quality-gated model because they either contained fewer than three Good-angle frames or failed to provide a valid Episode-level median dorsal ridge angle.

As shown in Figure 7, the confusion matrices were constructed by combining prediction results from all test Episodes that satisfied the quality-gating criteria under animal-wise three-fold cross-validation. Among the 151 original Episodes, 111 satisfied the quality-gating criteria and received predictions, whereas the remaining 40 Episodes did not produce predictions.

The five-class confusion matrix in Figure 7a shows that most predictions were concentrated along the diagonal and adjacent categories, although one extreme error occurred where a BCS 2 Episode was predicted as BCS 6. In the three-class management classification, no High-to-Lean misclassification was observed, although one Lean Episode was incorrectly predicted as High. Figure 7b shows that the High category achieved a recall of 1.0000, with no High Episodes misclassified as Lean. Additional extreme-error comparisons are provided in Appendix A (Figure A1).

### 3.4. Ablation Study Analysis

The ablation study results are presented in Table 8 and Figure 8. The contributions of different components to model performance varied across evaluation metrics. Coverage, Acc5, Macro-F1 5, QWK5, Within-one accuracy, and Macro-F1 3 are reported as the averaged results across the three-fold cross-validation.

First, under the condition of maintaining Low-condition and High-condition auxiliary supervision, the Acc5 values of the RGB-only LowHigh CO-MIL, Depth-only LowHigh CO-MIL, and RGB-depth LowHigh CO-MIL models were 0.2582, 0.3637, and 0.4562, respectively. The RGB-depth model outperformed both single-modality models on most metrics, including Acc5, Macro-F1 5, Within-one accuracy, and Macro-F1 3, supporting the complementary contributions of RGB appearance information and depth-based structural information. However, this advantage was not observed consistently across all evaluation metrics.

After introducing the Low-condition and High-condition auxiliary branches into the RGB-depth CO-MIL model, Macro-F1 5 increased from 0.3642 to 0.4069, whereas Acc5 decreased from 0.4763 to 0.4562, QWK decreased from 0.4197 to 0.3935, and Macro-F1 3 decreased from 0.6380 to 0.5842. These results indicate that auxiliary supervision may improve class-balance-related metrics, but its influence on overall classification performance is not uniformly positive across all evaluation criteria. Therefore, the Low-condition and High-condition branches mainly contribute to auxiliary feature learning rather than guaranteeing simultaneous improvement across all performance metrics.

When quality gating was introduced into the RGB-depth LowHigh CO-MIL model, Coverage decreased from 1.0000 to 0.7345, but prediction performance improved. Acc5 increased from 0.4562 to 0.5234, QWK increased from 0.3935 to 0.4800, and Macro-F1 3 increased from 0.5842 to 0.7188. These results suggest that, under the current dataset conditions, quality gating may reduce the influence of unreliable inputs on prediction and improve the stability of effective predictions among retained samples.

Under the same quality-gating criteria and identical eligible Episode set, further incorporating the Episode-level median dorsal ridge angle, Good-frame ratio, and transformed Good-angle-frame number led to additional performance improvements. Acc5 increased from 0.5234 to 0.6166, Macro-F1 5 increased from 0.4075 to 0.5680, QWK increased from 0.4800 to 0.6339, and Macro-F1 3 increased from 0.7188 to 0.8059. Within-one accuracy showed only a slight change, decreasing from 0.9022 to 0.8971. These original results concern the combined angle and quality-statistic feature set and cannot isolate the contribution of the angle itself; the separate component results are reported in Section 3.7.

The Angle-only MLP model achieved Acc5 and Macro-F1 5 values of 0.1990 and 0.1637, respectively, further demonstrating that low-dimensional angle features alone are insufficient for stable five-class BCS prediction.

Overall, RGB-D multimodal information, the quality-gating strategy, and Episode-level geometric features contribute to the final model performance from three complementary perspectives: visual information fusion, input reliability control, and biologically meaningful structural representation.

### 3.5. Matched-Subset Analysis of Quality Gating Effect

To exclude the influence of performance differences caused by different evaluation sets, an additional matched-subset analysis was conducted. This analysis selected the 111 test Episodes that satisfied the quality-gating criteria and were eligible for prediction. The non-gated RGB-depth LowHigh CO-MIL model and the quality-gated RGB-depth LowHigh model without angle features were compared using exactly the same Episode set.

As shown in Table 9, under this controlled comparison condition, introducing quality gating increased the Acc5 from 0.5005 to 0.5234, QWK5 from 0.4462 to 0.4800, and Within-one accuracy from 0.7810 to 0.9022 in the five-class BCS classification task. For the three-class body condition classification task, Acc3 increased from 0.6114 to 0.8154, Macro-F1 3 increased from 0.6124 to 0.7188, and QWK3 increased from 0.5414 to 0.6279. Meanwhile, Macro-F1 5 slightly changed from 0.4128 to 0.4075 and did not show a clear improvement.

These results indicate that, under the same test Episode conditions, the performance differences introduced by quality gating were not solely attributable to reducing the number of predicted samples or changing the evaluation set. Quality gating improved several prediction performance metrics, particularly in terms of prediction error control and three-class body condition classification. However, its effects were not consistent across all metrics, and the improvement for fine-grained five-class classification remained relatively limited.

### 3.6. Sensitivity Analysis and Robustness of Quality Control

#### 3.6.1. Sensitivity to the Good-Angle Frame Threshold $\tau_{frame}$

As shown in Table 10, to determine the Episode eligibility threshold $\tau_{frame}$ (the minimum number of Good-angle frames required per Episode), a sensitivity analysis was conducted by scanning candidate values {1, 2, 3, 4, 5} and evaluating their effects on Coverage and the performance of the Final model.

Coverage decreased monotonically with increasing $\tau_{frame}$ (0.9470 → 0.8609 → 0.7351 → 0.6093 → 0.4967), accompanied by a reduction in the number of qualified Episodes (143 → 130 → 111 → 92 → 75). Classification performance exhibited an inverted-U trend and reached a plateau when $\tau_{frame}$ ≥ 3. Acc5 increased from 0.5880 at $\tau_{frame}$ = 1 to 0.6166 at $\tau_{frame}$ = 3 and 0.6210 at $\tau_{frame}$ = 4, followed by a slight decrease to 0.6188 at $\tau_{frame}$ = 5. The trend of Macro-F1 5 was consistent with Acc5. The difference in Acc5 among $\tau_{frame}$ = 3, 4, and 5 was only 0.0044, indicating that further increasing $\tau_{frame}$ provided negligible marginal gains in classification performance.

Considering the Coverage–performance trade-off, $\tau_{frame}$ = 3 was selected as the default threshold. Its Acc5 (0.6166) differed from the global optimum ($\tau_{frame}$ = 4, 0.6210) by only 0.0044, while its Coverage was 23.84 percentage points higher than that of $\tau_{frame}$ = 5 (111 vs. 75 Episodes). Therefore, a nearly negligible loss in accuracy allowed substantially more predictable samples to be retained, which better meets practical deployment requirements.

From a biological perspective, BCS 4–5 accounted for 66.89% of all Episodes. An overly strict threshold ($\tau_{frame}$ ≥ 4 would remove a considerable proportion of these dominant classes (the retention rate of BCS 4 decreased from 0.66 to 0.42), potentially aggravating class imbalance. The selected threshold $\tau_{frame}$ = 3 maintained an average of 16.34 valid frames per Episode while preserving the original data distribution as much as possible, and its performance was fully consistent with the final results reported.

Clarification of evaluation criteria: Metrics under different $\tau_{frame}$ values were calculated on their respective eligible Episode sets, meaning that denominators differed. Therefore, Coverage is the primary indicator reflecting the strictness of quality gating. The potential selection bias introduced by filtering low-quality inputs was addressed through the matched-subset analysis using the same 111 Episodes.

#### 3.6.2. Within-Episode Angle Consistency

The Median Absolute Deviation (MAD) of all valid cross-sectional angles from Good-angle frames within the same Episode was calculated: MAD=1.340°.

This result indicates high consistency of angle estimation for the same animal during short-term continuous acquisition. If the geometric parameters (dorsal ridge vertex within 35–65% of body width, bilateral contour points within 5–40%, and at least four points per side) were highly sensitive, small variations in posture or viewing angle within the same Episode would be expected to introduce larger fluctuations. However, the measured MAD was only 1.340°, suggesting that angle estimation showed relatively low variability under the current acquisition conditions.

This MAD describes angle-measurement consistency only; prediction-level evidence is evaluated separately in Section 3.7.

#### 3.6.3. Fitting Quality and Outlier Rejection

To further evaluate the ability of the quality-gating mechanism to handle frame-level fitting imperfections, the distribution of fitting quality $R^{2}$ values for all qualified cross-sections was analyzed, as shown in Table 11.

Among 15,601 cross-sections, the mean $R^{2}$ averaged across the left and right fitted profiles was 0.913, with a median value (q50) of 0.949. Only 8.03% of cross-sections had $R^{2}$ < 0.80. Across BCS categories, BCS 5 showed the highest fitting quality (mean $R^{2}$ = 0.943, with 3.16% below 0.80), whereas BCS 6 showed the lowest fitting quality (mean $R^{2}$ = 0.886, with 13.06% below 0.80), possibly because high-condition animals exhibit smoother dorsal contour variations.

Based on the above distribution, the effect of introducing an additional hard exclusion threshold of $R^{2}$ < 0.80 was simulated. After removing cross-sectional angles below this threshold from the Episode-level angle sets and recalculating the Episode-level median angles, the pooled absolute change in median angle was only approximately 0.112°. Among the 111 eligible Episodes, only 3 Episodes (2.7%) showed changes exceeding 1°.

### 3.7. Additional Component and Episode Prediction Results

Table 12 summarizes the additional matched experiments. Adding angle to quality statistics changed Acc5 by only 0.0060 (VAQ − VQ; 95% CI −0.0218 to 0.0370). Without quality statistics, the angle increment was 0.0210 (VA − V; −0.0249 to 0.0673); the quality-statistic increment without angle was also 0.0210 (VQ − V; −0.0262 to 0.0692). These intervals include zero, so an independent predictive benefit of the angle was not established. The attention model V exceeded the single-frame control by 0.0571 Acc5 (0.0060 to 0.1143) and mean pooling by 0.0511 (0.0190 to 0.0887), providing exploratory support for multi-frame aggregation and attention pooling.

Values are pooled out-of-fold metrics (111 Episodes per seed), summarized across three seeds. V, VA, VQ and VAQ are defined in Section 2.4.3; V_single and V_mean use single-frame and mean-pooling visual inputs. Unlike Table 7, Table 8 and Table 9, SD here describes seed variation, not variation across folds.

For VAQ, agreement with the deterministic reference prediction increased from 0.9025 with one sampled frame to 0.9602 with 12 (95% CI 0.9437–0.9753); mean absolute BCS change decreased from 0.1166 to 0.0423. At 12 sampled frames, accuracy against the reference BCS was 0.5714 (0.5030–0.6470), demonstrating that high consistency does not imply equally high accuracy. The mean number of unique frames was 7.83 because some Episodes required replacement sampling.

Both halves independently passed quality gating for 59 Episodes (53.15% of the 111 full-eligible Episodes; 39.07% of all 151). Across seeds, VAQ exact agreement between halves ranged from 0.9322 to 0.9831 (mean 0.9548), and all paired predictions differed by at most one BCS category. Mean first-half, second-half and full-Episode accuracies on these same 59 Episodes were 0.5989, 0.6102 and 0.6215, respectively. Thus, the results support conditional within-Episode prediction consistency, with limited coverage and moderate classification accuracy.

## 4. Discussion

This study proposed an Episode-level RGB-D video-based framework for automated cattle BCS, integrating multi-frame visual information aggregation, geometric quality gating, and biologically meaningful dorsal morphological features to improve the reliability of automated BCS assessment under complex acquisition conditions. Unlike conventional approaches based on single-frame image classification, this study defined the RGB-D video segment collected from the same animal during one continuous acquisition process as an Episode and aggregated multiple instances using Attention-MIL [18,19]. This design is consistent with the biological characteristics of BCS as an indicator of overall animal nutritional status and energy reserves, because BCS is not determined by a single instantaneous posture but reflects the overall body shape and fat deposition status of the animal.

During Episode-level modeling, each video frame was considered as an instance in MIL, and the attention mechanism was used to learn the relative importance of different frames for the final prediction. It should be noted that Attention-MIL does not represent an explicit selection of manually defined optimal observation frames. Instead, its primary function is to integrate observations with different postures, viewpoints, and quality levels during continuous acquisition, thereby reducing the influence of individual abnormal frames on the final prediction. In addition, using Episode as the fundamental prediction unit prevents highly correlated frames collected from the same animal within a short period from being treated as completely independent samples, thereby reducing the risk of potential data leakage [16,17]. For animal video data with strong temporal correlation, Episode-level modeling is more consistent with practical monitoring scenarios than frame-level prediction.

The quality-gating mechanism introduced in this study was designed to address the instability of RGB-D data quality under practical acquisition conditions. Unlike the classification model, quality gating was not optimized using BCS labels but instead evaluated input reliability based on geometric criteria, including trunk completeness, posture plausibility, dorsal contour continuity, and angle computability. Only Episodes containing a sufficient number of Good-angle frames and providing reliable Episode-level dorsal angle estimation were included in the final prediction set. The final model achieved an average Coverage of 0.7345, indicating that quality gating could reduce the potential influence of unreliable inputs on the prediction process to some extent. However, quality gating also resulted in some Episodes not receiving predictions. Therefore, it should be considered as a selective prediction strategy that balances prediction reliability and coverage, rather than simply a method for increasing classification accuracy. Similar selective prediction strategies have been investigated in machine learning to allow models to abstain from uncertain predictions when input reliability is insufficient [29].

It should be particularly emphasized that during the initial data organization stage, this study did not perform sample selection based on BCS category, animal posture, dorsal morphology, or prediction difficulty. After parsing the original dataset, 5940 synchronized RGB-D frame pairs were obtained. Among them, 2125 frame pairs were removed because they did not contain valid cattle subjects (e.g., frames without an animal target), and the remaining 3815 valid synchronized RGB-D frame pairs were retained for subsequent analysis. This step was only performed to remove data that could not provide animal observations and was not part of the proposed geometric quality-gating procedure. Subsequently, filtering related to posture variation, occlusion, depth quality, and dorsal contour reliability was conducted by the proposed quality-gating module. Therefore, the basic data cleaning process and model-stage quality control represent two clearly distinct procedures.

This study further investigated the relationship between dorsal ridge angle and BCS. The statistical analysis showed that Episode-level dorsal ridge angle exhibited a moderate positive correlation with BCS categories, and significant differences in angle distributions were observed among different BCS classes. These findings indicate that dorsal geometric information extracted from the Depth Map contains body-condition-related structural information. This result is consistent with the characteristics of manual BCS assessment, which considers dorsal contour, skeletal prominence, and changes in fat coverage [7,11,12]. However, it should be emphasized that the dorsal ridge angle calculated in this study was derived from normalized two-dimensional depth profiles rather than fully calibrated three-dimensional anatomical parameters. Therefore, this angle is not a direct measurement of fat thickness or fat deposition, but rather an external morphological descriptor associated with body condition variation.

The Angle-only MLP experiment further demonstrated that the three-variable geometric feature set was insufficient for stable five-class BCS prediction. Although Episode-level dorsal angle and its reliability statistics reflected part of the body condition variation, BCS changes involve multiple anatomical regions and complex body structural variations. Therefore, relying solely on these dorsal geometry and quality features cannot fully represent the complete evaluation process. The angle-related features are more appropriately considered complementary information to RGB-D visual representations rather than independent prediction factors. When geometric features were fused with RGB-D visual information, model performance further improved, suggesting that explicit structural information can provide additional biological constraints for end-to-end visual feature learning.

The matched component analysis did not establish an independent angle benefit after controlling for quality statistics and model capacity. Its association with BCS should therefore not be equated with incremental predictive utility. The additional prediction tests support within-Episode consistency, but moderate accuracy and restricted both-half eligibility preclude a claim of generally reliable BCS decisions. The original High recall of 1.0000 also did not hold in every additional run (VAQ range 0.9565–1.0000).

The ablation experiments demonstrated that different components played distinct roles within the final framework. The RGB-depth model outperformed RGB-only and Depth-only models on most evaluation metrics, indicating complementary contributions between RGB appearance information and Depth-based spatial structural information [13,14]. RGB information provides surface visual characteristics of the animal, whereas Depth information supplements trunk spatial structure and contour variation. Their fusion helps reduce the limitations caused by insufficient information from a single modality.

For the Low-condition and High-condition auxiliary branches, experimental results showed that their effects were metric-dependent. Although auxiliary supervision improved some class-balance-related metrics, it did not result in simultaneous improvements across all evaluation metrics. Therefore, the current results do not support the conclusion that auxiliary heads provide additional supervision for extreme-condition representation learning. A more appropriate interpretation is that the auxiliary branches serve as additional training constraints by providing supervision signals related to extreme body conditions, which may facilitate feature learning, while the final prediction capability remains primarily dependent on the five-class BCS classification task.

In recent years, automated BCS assessment methods based on computer vision and deep learning have continued to develop, with different studies exploring RGB images, depth information, and video sequence modeling [6,12,13]. More recent studies have further explored deep learning-based approaches using RGB and RGB-depth data [30,31]. However, direct numerical comparison among studies remains difficult due to differences in data sources, acquisition environments, scoring systems, prediction units, and validation strategies. The primary contribution of this study is not solely pursuing the highest classification accuracy, but exploring an integrated BCS assessment framework that combines multi-frame information, quality control, and interpretable geometric features under conditions involving video quality variation, limited sample size, and practical deployment constraints.

Several limitations remain in this study. First, the current dataset was collected from a single Raramuri Criollo herd and a single acquisition environment. Therefore, the generalization capability of the proposed model requires further evaluation through external validation involving different breeds, production systems, and farm environments. Differences in body structure, coat characteristics, management practices, and acquisition devices may influence model performance.

Second, the dataset size was relatively limited, containing only 151 Episodes and 52 Animal identity groups. Although the Animal-wise split prevented related samples from the same animal from simultaneously appearing in training and testing sets, the limited sample size may still lead to performance variation among different folds, particularly for minority categories. The small number of BCS 2 samples limits reliable recognition of extremely low-condition animals. Future studies should collect more extreme-condition samples to improve learning capability for boundary categories.

Third, the quality-gating threshold was determined through sensitivity analysis. The results showed that increasing the Good-angle frame threshold could slightly improve some classification metrics but substantially reduce Coverage. Therefore, $\tau_{frame}$ = 3 was selected as a compromise between prediction reliability and coverage. Future studies could explore continuous quality scoring, adaptive thresholds, or learning-based quality assessment methods to further optimize coverage.

Fourth, although Depth information provides rich three-dimensional structural information, this study did not explicitly detect specific anatomical landmarks traditionally considered in BCS assessment, such as lumbar spinous and transverse processes, hooks, pins, and the tail-head region. The dorsal ridge angle used in this study mainly describes overall dorsal contour variation. Future studies could further integrate three-dimensional keypoint detection, local curvature analysis, and region-level geometric features to better exploit anatomical information provided by RGB-D sensors and investigate the contribution of different body regions to BCS prediction.

In addition, the current angle-fitting procedure did not incorporate independent point-level outlier removal or a hard goodness-of-fit threshold. Although the existing quality-control procedure can already filter some unreliable contours, future work could combine robust regression, confidence-weighted fitting, or automatic outlier detection methods to improve the stability of geometric feature extraction under depth noise conditions.

Finally, this study mainly validated the effectiveness of the Episode-level prediction framework under controlled acquisition conditions and has not yet systematically evaluated long-term stability under cross-scenario deployment. Future studies should incorporate independent farm datasets, multi-Episode sequential decision strategies, and richer three-dimensional anatomical features to further improve applicability in real production environments.

Overall, this study demonstrates that integrating RGB-D multimodal information, Episode-level multi-frame modeling, and biologically meaningful geometric features provides a promising technical pathway for automated cattle BCS monitoring. However, the proposed method should currently be considered an auxiliary assessment tool under controlled conditions rather than a complete replacement for professional BCS evaluation. By expanding dataset size, strengthening external validation, and further exploiting three-dimensional anatomical information, future research may facilitate the transition of automated BCS monitoring methods toward practical production applications.

## 5. Conclusions

This study proposed an Episode-level body condition scoring framework for beef cattle that integrates multi-frame RGB-D information, Attention-MIL, geometry-based quality gating, and biologically relevant dorsal morphological features. Under Animal-wise three-fold cross-validation, the final model achieved a five-class accuracy of 0.6166, a Macro-F1 score of 0.5680, a quadratic weighted kappa of 0.6339, and a Within-one accuracy of 0.8971 at a mean Coverage of 0.7345. After mapping the predictions into three body condition categories (Lean, Ideal, and High), the model achieved a Macro-F1 score of 0.8059.

The results indicate that RGB and Depth information provide complementary representations, while the original ablation supports the combined angle and quality-statistic feature set. The additional matched experiments support multi-frame aggregation and conditional within-Episode consistency, but do not establish an independent predictive benefit of the angle. The geometry-based quality-gating strategy functions as a selective-prediction mechanism by withholding predictions when sufficient geometric evidence is unavailable, thereby balancing prediction reliability and coverage. Overall, this study demonstrates the potential of combining multimodal RGB-D information, Episode-level modeling, and interpretable geometric features for automated BCS assessment in beef cattle under controlled acquisition conditions.

However, the current evaluation was conducted using a relatively small dataset from a single Raramuri Criollo herd, and external validation across different breeds and farm environments is still required before practical deployment. Future studies should further investigate cross-farm generalization, richer anatomical feature extraction from depth information, and larger datasets containing more extreme body condition categories.

## Figures and Tables

**Figure 1:**
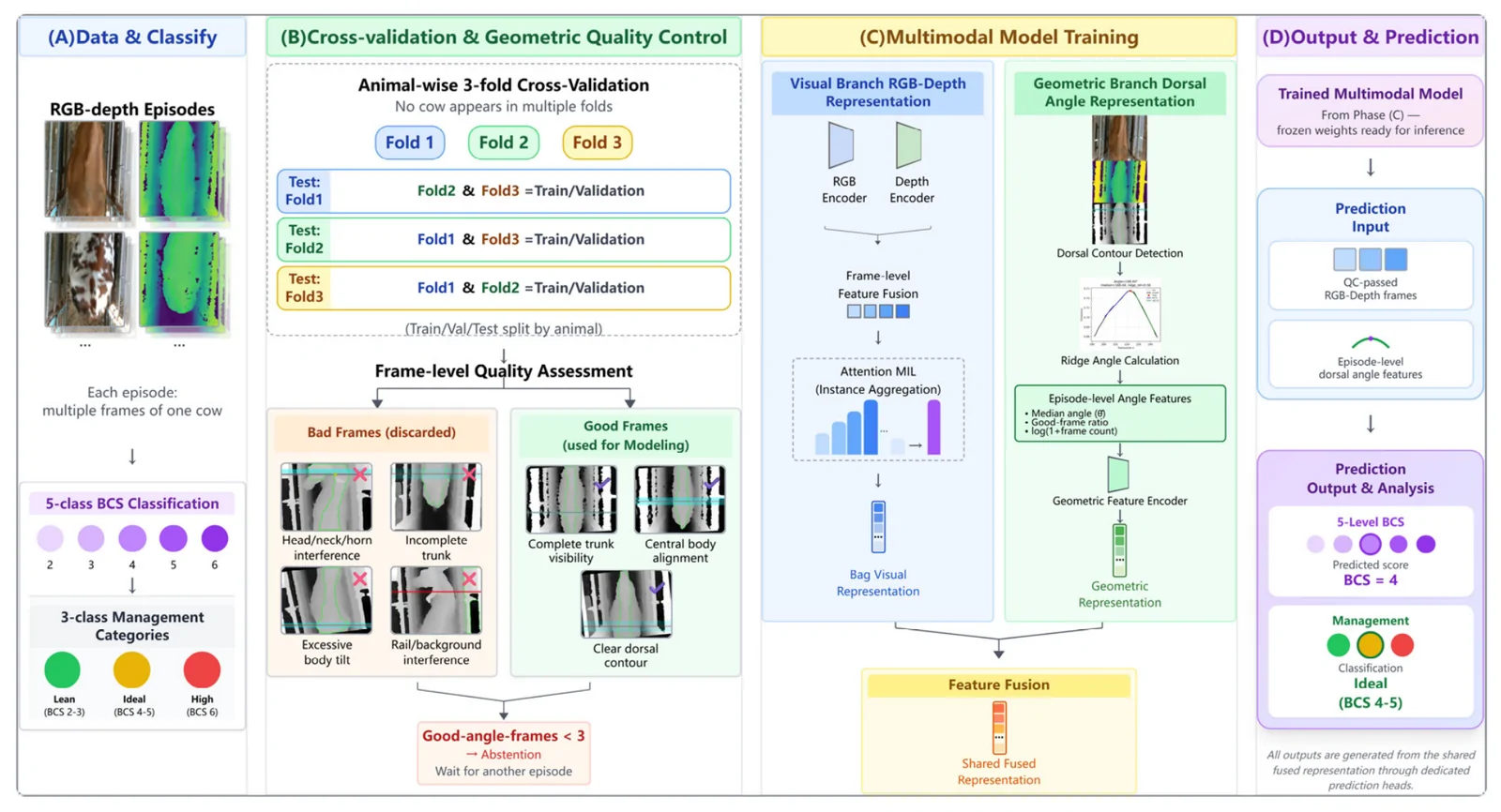
Overall workflow of the proposed framework.

**Figure 2:**
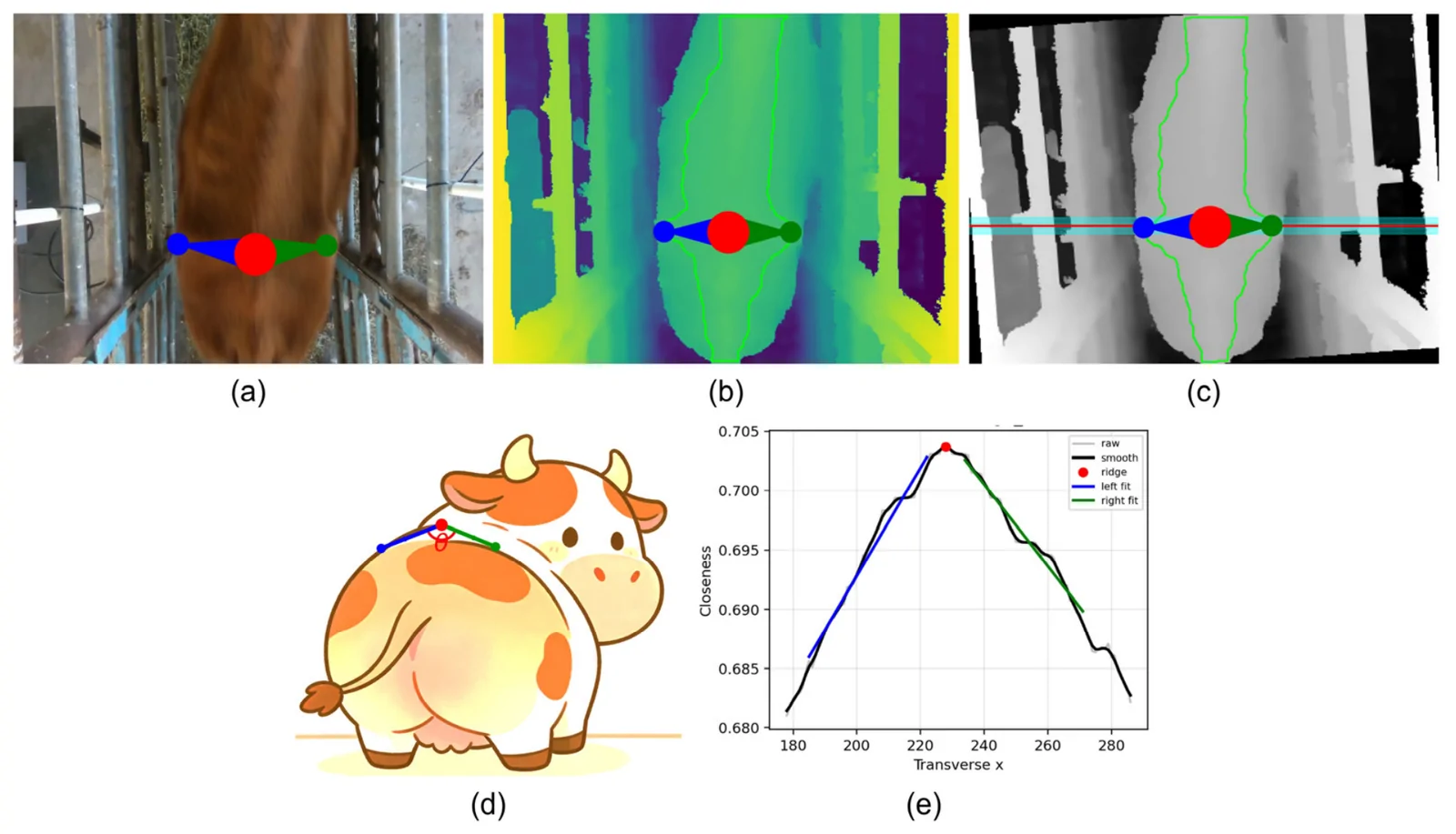
Schematic illustration of dorsal ridge angle extraction. (**a**) RGB image; (**b**) Pseudocolor depth image and candidate trunk contour; (**c**) Grayscale depth image with sampled cross-sections; (**d**) Schematic dorsal ridge angle; (**e**) Fitted normalized depth profile. Green contours outline the trunk; cyan and red lines in (**c**) mark sampled and illustrated cross-sections. Red points indicate the ridge vertex, and blue and green lines denote the left and right profiles; θ denotes the included angle. In (**e**), gray and black curves show the raw and smoothed profiles, respectively.

**Figure 3:**
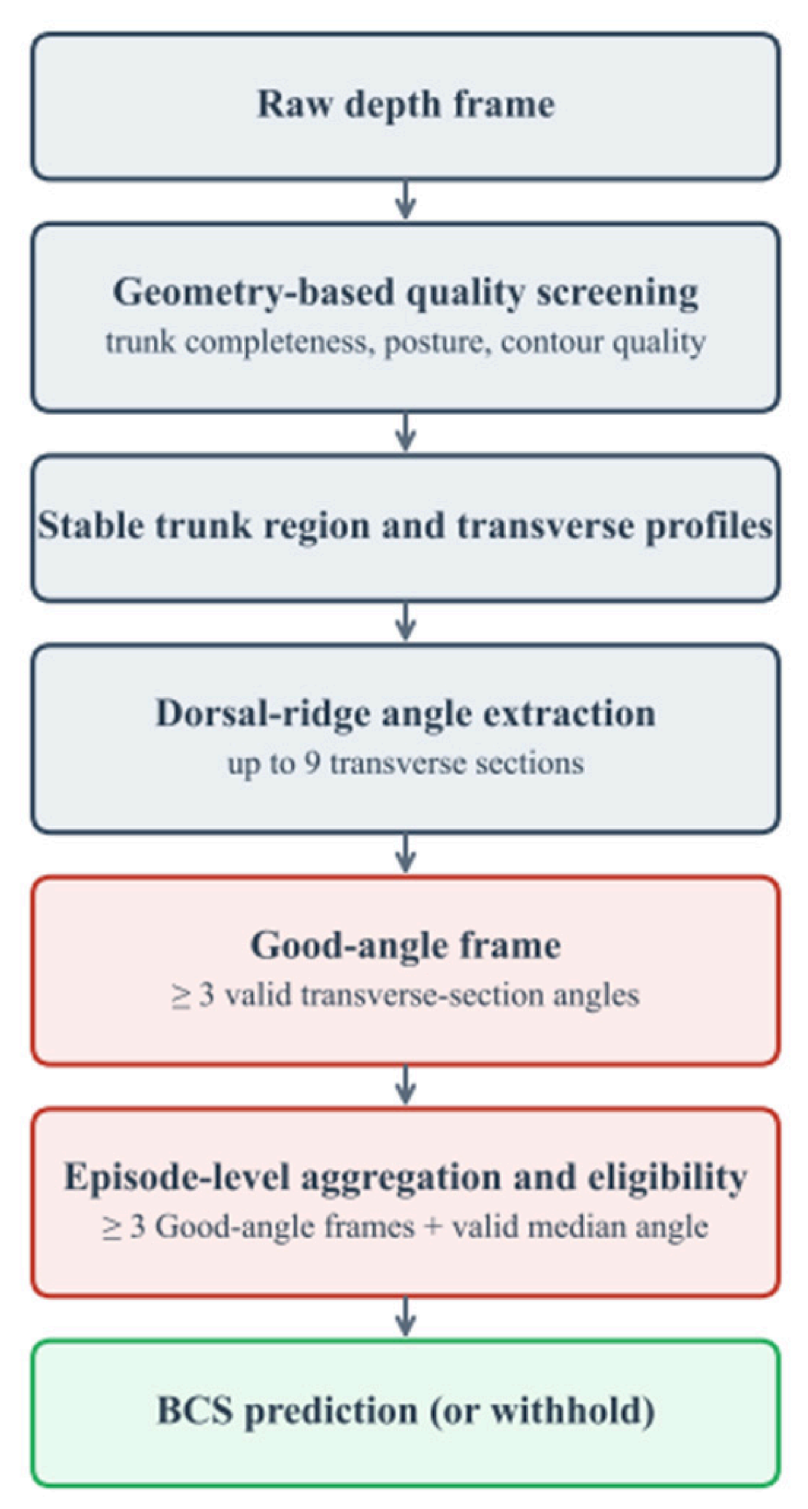
Simplified workflow of geometric quality control and dorsal ridge angle extraction.

**Figure 4:**
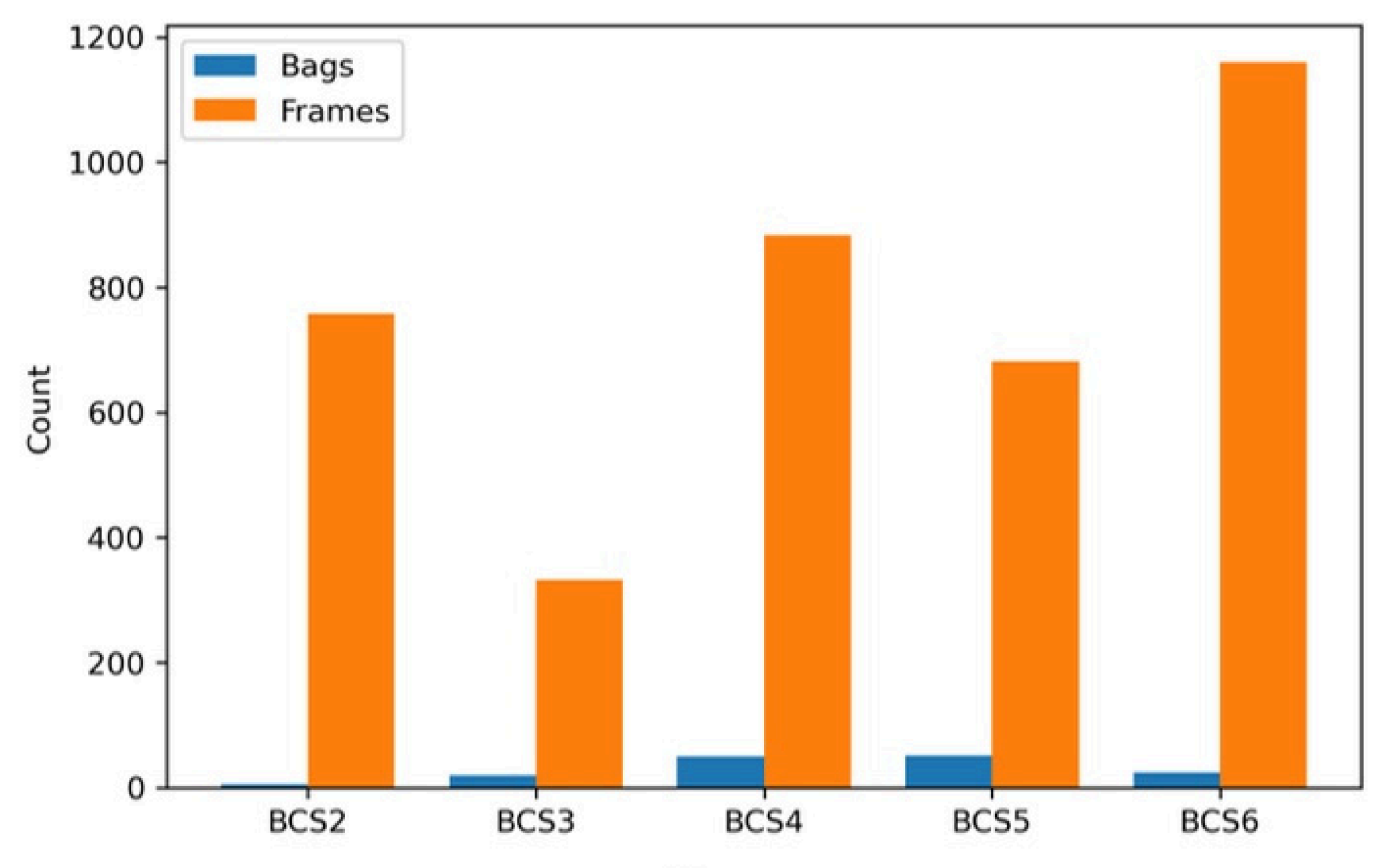
Class distribution of the dataset.

**Figure 5:**
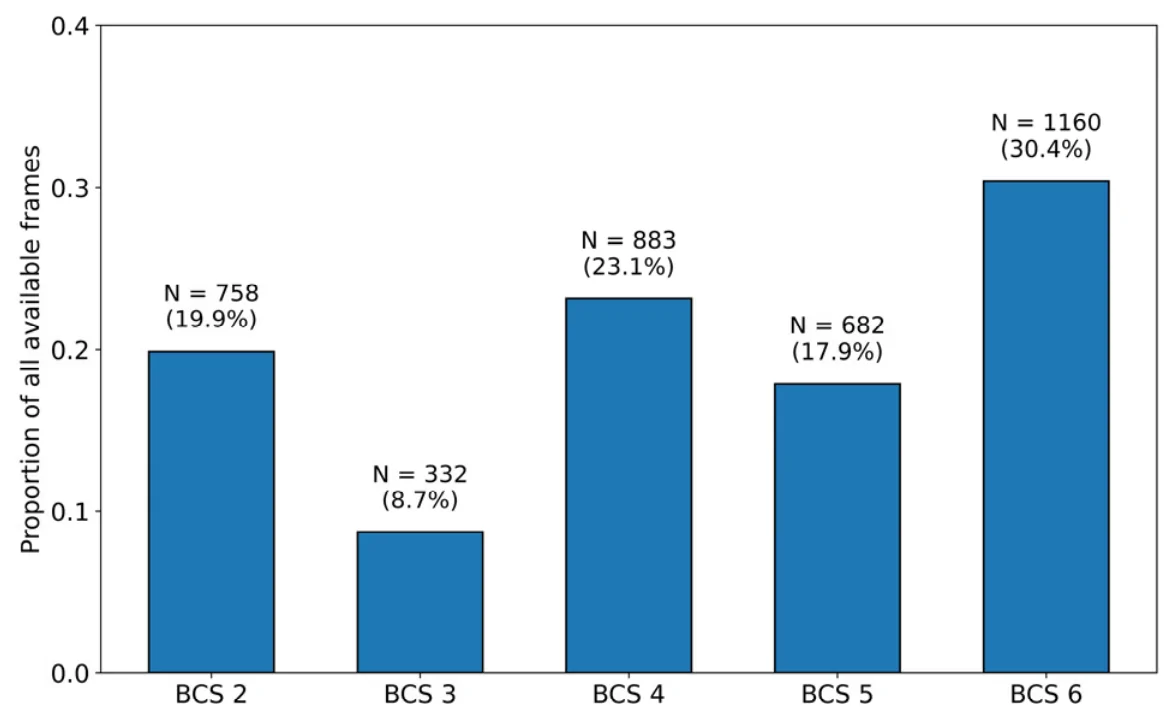
Distribution of available frames across BCS classes.

**Figure 6:**
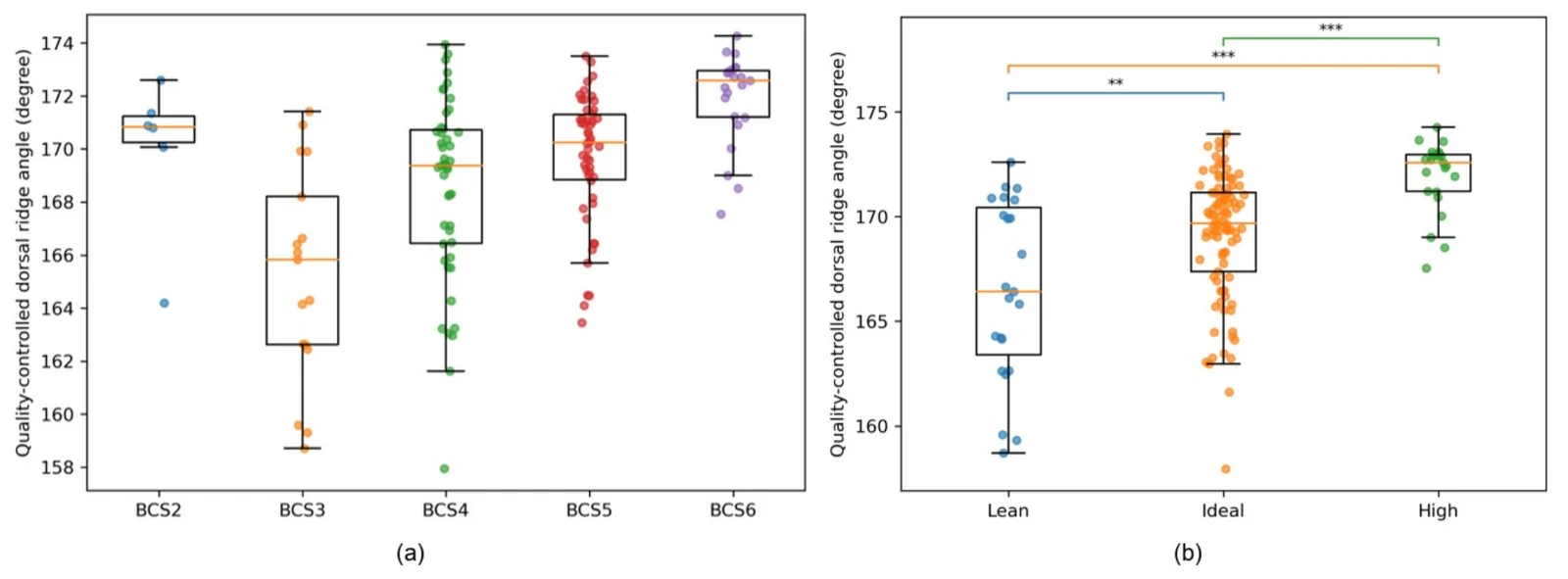
Distribution of Episode-level dorsal ridge angles across different BCS categories and management classes. (**a**) Angle distribution across BCS 2–6; (**b**) Angle distribution across three management classes (Lean, Ideal, High). ** adjusted *p* < 0.01; *** adjusted *p* < 0.001, following Benjamini–Hochberg correction.

**Figure 7:**
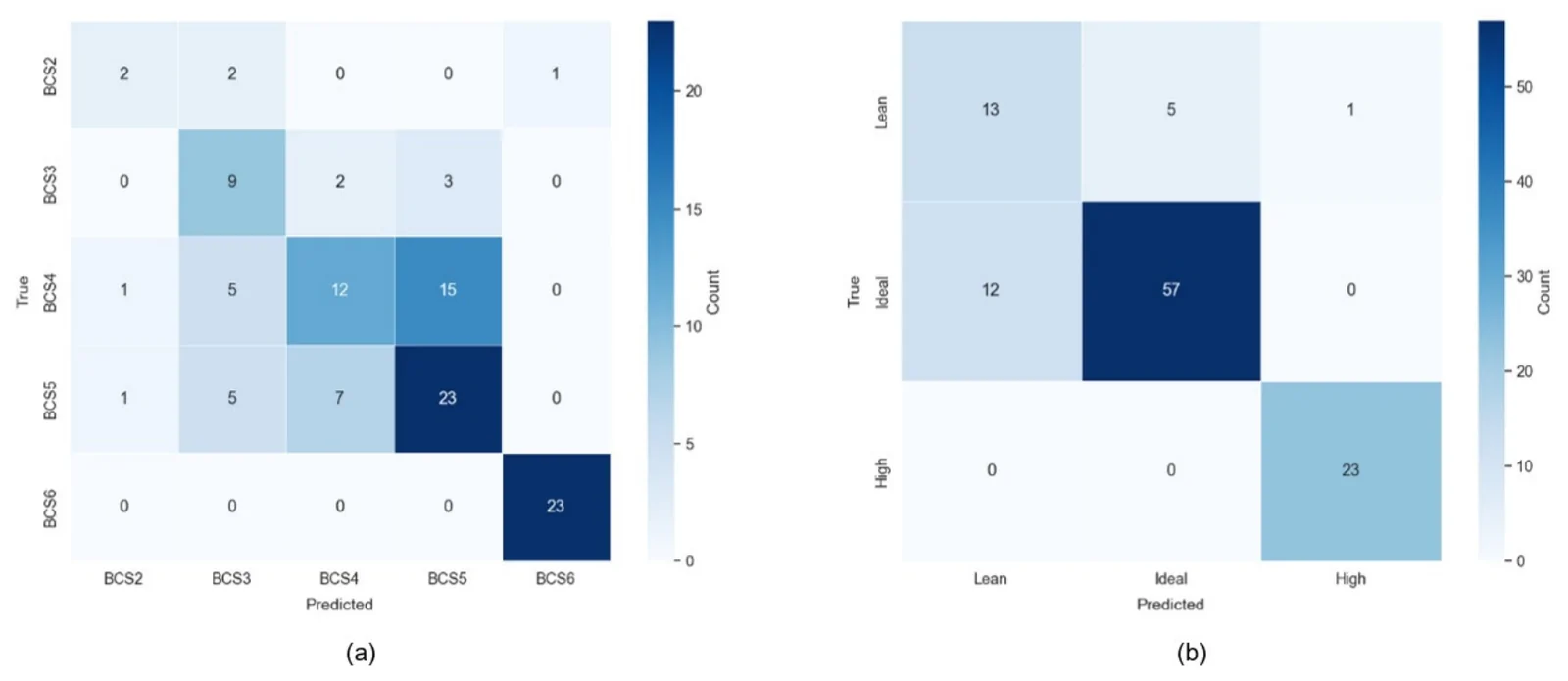
Confusion matrices of the final model based on pooled out-of-fold predictions. (**a**) Five-class BCS classification; (**b**) Three-category body condition classification of Lean, Ideal, and High.

**Figure 8:**
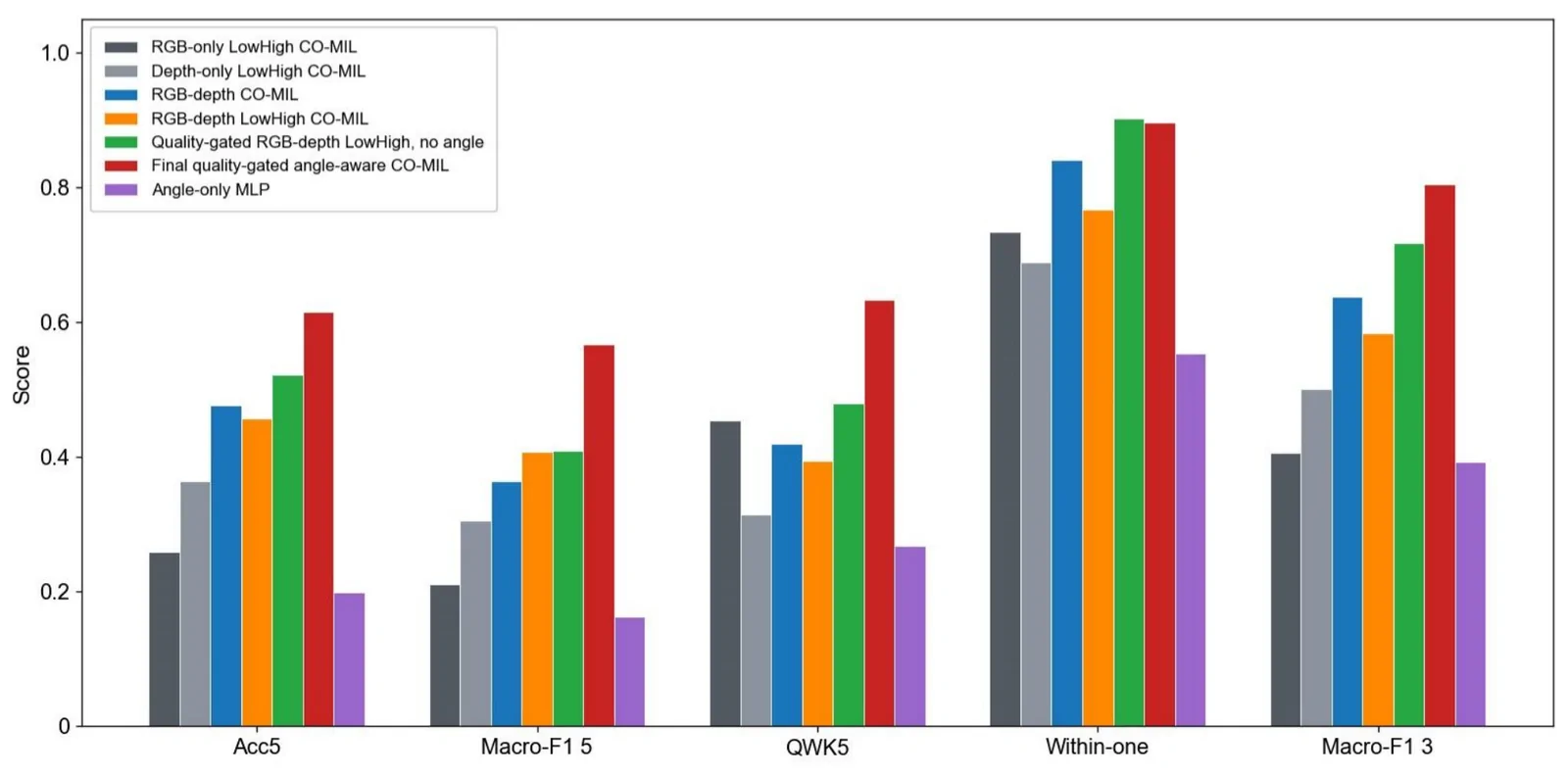
Main performance metrics of the different model variants.

**Table 1:** Summary of dataset preprocessing and final analytical dataset.

Item	Value
Original synchronized RGB-D frame pairs	5940
Invalid frame pairs removed during data validation	2125
RGB-D frame pairs retained after preprocessing	3815
Total episodes	151
Animal identity grouping identifiers used for split control	52
BCS 2 episodes	6
BCS 3 episodes	20
BCS 4 episodes	50
BCS 5 episodes	51
BCS 6 episodes	24
BCS 2 frames	758
BCS 3 frames	332
BCS 4 frames	883
BCS 5 frames	682
BCS 6 frames	1160

**Table 2:** Design and purpose of the model variants used in the ablation study.

Model	RGB	Depth Map	Low/High-Condition Auxiliary	Quality Gate	Angle-Related Geometric Features
1. RGB-only LowHigh CO-MIL	✓		✓		
2. Depth-only LowHigh CO-MIL		✓	✓		
3. RGB-depth CO-MIL	✓	✓			
4. RGB-depth LowHigh CO-MIL	✓	✓	✓		
5. Quality-gated RGB-depth LowHigh, no angle	✓	✓	✓	✓	
6. Final quality-gated angle-aware CO-MIL	✓	✓	✓	✓	✓
7. Angle-only MLP				✓	✓

✓ indicates that the component is included; a blank cell indicates that it is not included.

**Table 3:** Episode distribution of the test sets under Animal-wise three-fold cross-validation.

Fold	Fold 1	Fold 2	Fold 3	Total
Raw test bags	51	50	50	151
Animal identity group	18	17	17	52
BCS2 bags	1	3	2	6
BCS3 bags	10	5	5	20
BCS4 bags	15	17	18	50
BCS5 bags	16	18	17	51
BCS6 bags	9	7	8	24

**Table 4:** Frame-level geometric quality control statistics across BCS classes.

BCS	Available Frames	Frame-Level Pass Rate	Proportion of All Available Frames	Median Good-Angle-Frame Number per Episode
2	758	49.1%	19.9%	4.2
3	332	48.2%	8.6%	4.0
4	883	48.7%	23.1%	5.1
5	682	49.0%	17.9%	5.3
6	1160	49.1%	30.5%	6.0
Total	3815	48.9%	—	—

This statistic reflects differences in the number of Good-angle frames among different BCS categories and provides descriptive information for subsequent analysis of angle feature reliability.

**Table 5:** Statistical analysis of Episode-level dorsal ridge angles across BCS classes.

Comparison	Test Statistic	*p*-Value	Adjusted *p*-Value	Effect Size
Spearman correlation (BCS vs. angle)	ρ = 0.440	<0.001	<0.001	—
Kruskal–Wallis across BCS classes	H = 39.107	<0.001	<0.001	—
Lean vs. Ideal	U = 719	0.0083	0.0083	Cliff’s δ = −0.355
Lean vs. High	U = 49	<0.001	<0.001	Cliff’s δ = −0.815
Ideal vs. High	U = 424	<0.001	<0.001	Cliff’s δ = −0.620

**Table 6:** Coverage and number of eligible Episodes.

Fold	Test the Number of Episodes	Qualified Episodes	Coverage
Fold 1	51	42	0.8235
Fold 2	50	36	0.7200
Fold 3	50	33	0.6600
Mean ± SD across folds	151	111	0.7345 ± 0.0827
Pooled eligible episodes:			0.7351

**Table 7:** Performance of the Final quality-gated angle-aware CO-MIL model.

Model	Final Quality-Gated Angle-Aware CO-MIL
Coverage	0.7345 ± 0.0827
Acc5	0.6166 ± 0.1208
Macro-F1 5	0.5680 ± 0.1504
Weighted-F1 5	0.5952 ± 0.1430
QWK5	0.6339 ± 0.2865
MAE	0.5166 ± 0.2397
Within-one	0.8971 ± 0.0688
Acc3	0.8367 ± 0.0506
Macro-F1 3	0.8059 ± 0.0816
QWK3	0.7603 ± 0.1300
Lean recall	0.6667 ± 0.1667
High recall	1

**Table 8:** Main ablation results for the different model variants.

Variant	Coverage	Acc5	Macro-F1 5	QWK5	Within-One	Macro-F1 3
RGB-only LowHigh CO-MIL	1	0.2582	0.2103	0.4530	0.7350	0.4051
Depth-only LowHigh CO-MIL	1	0.3637	0.3047	0.3142	0.6886	0.5022
RGB-depth CO-MIL	1	0.4763	0.3642	0.4197	0.8412	0.6380
RGB-depth LowHigh CO-MIL	1	0.4562	0.4069	0.3935	0.7676	0.5842
Quality-gated RGB-depth LowHigh, no angle	0.7345	0.5234	0.4075	0.4800	0.9022	0.7188
Final quality-gated angle-aware CO-MIL	0.7345	0.6166	0.5680	0.6339	0.8971	0.8059
Angle-only MLP	0.7345	0.1990	0.1637	0.2674	0.5548	0.3922

**Table 9:** Matched-subset performance comparison of non-gated and quality-gated RGB-depth LowHigh CO-MIL models.

Model	RGB-Depth LowHigh CO-MIL, Matched 111	Quality-Gated RGB-Depth LowHigh, No Angle
Acc5	0.5005 ± 0.2410	0.5234 ± 0.0970
Macro-F1 5	0.4128 ± 0.1454	0.4075 ± 0.1024
QWK5	0.4462 ± 0.1908	0.4800 ± 0.0466
MAE	0.8312 ± 0.4385	0.6390 ± 0.0752
Within-one	0.7810 ± 0.1712	0.9022 ± 0.0188
Acc3	0.6114 ± 0.2916	0.8154 ± 0.0591
Macro-F1 3	0.6124 ± 0.2294	0.7188 ± 0.1078
QWK3	0.5414 ± 0.2038	0.6279 ± 0.0689

All results are presented as mean ± standard deviation across three-fold cross-validation. Both models were evaluated on exactly the same 111 test Episodes that satisfied the quality-gating prediction criteria.

**Table 10:** Sensitivity of Episode-level quality gating to the minimum Good-angle frame threshold $\tau_{frame}$.

$\tau_{frame}$	Qualified Episodes	Coverage	Acc5	Macro-F1 5
1	143	0.9470	0.5880	0.5312
2	130	0.8609	0.6025	0.5520
**3**	**111**	**0.7351**	**0.6166**	**0.5680**
4	92	0.6093	0.6210	0.5715
5	75	0.4967	0.6188	0.5690

The Final quality-gated angle-aware CO-MIL model was evaluated under each threshold while all other settings were kept unchanged. The default threshold $\tau_{frame}$ is highlighted in bold. Coverage in this sensitivity analysis was calculated as the pooled number of eligible Episodes divided by all 151 Episodes, whereas model-level Coverage reported elsewhere in the manuscript was calculated as the mean value across the three test folds.

**Table 11:** Distribution of cross-section fitting $R^{2}$ by BCS class (*n* = 15,601 profiles).

BCS	*n*_Profiles	Mean R^2^	q05	q25	q50 (Median)	q75	Frac_Below_0.80
2	1515	0.904	0.747	0.893	0.938	0.964	0.0805
3	1132	0.924	0.786	0.900	0.946	0.973	0.0627
4	3744	0.930	0.801	0.918	0.955	0.976	0.0494
5	3321	0.943	0.846	0.932	0.961	0.978	0.0316
6	5889	0.886	0.546	0.877	0.938	0.972	0.1306
ALL	15,601	0.913	0.734	0.904	0.949	0.974	0.0803

**Table 12:** Additional matched component and aggregation results.

Variant	Acc5	Macro-F1 5	QWK5
V	0.5526 ± 0.0290	0.5089 ± 0.0466	0.5678 ± 0.0754
VA	0.5736 ± 0.0138	0.5115 ± 0.0242	0.5996 ± 0.0522
VQ	0.5736 ± 0.0375	0.5361 ± 0.0355	0.6015 ± 0.0349
VAQ	0.5796 ± 0.0496	0.5313 ± 0.0691	0.6012 ± 0.0623
V_single	0.4955 ± 0.0238	0.4475 ± 0.0443	0.5345 ± 0.0958
V_mean	0.5015 ± 0.0462	0.4475 ± 0.0722	0.5109 ± 0.0492

**Figure A1:**
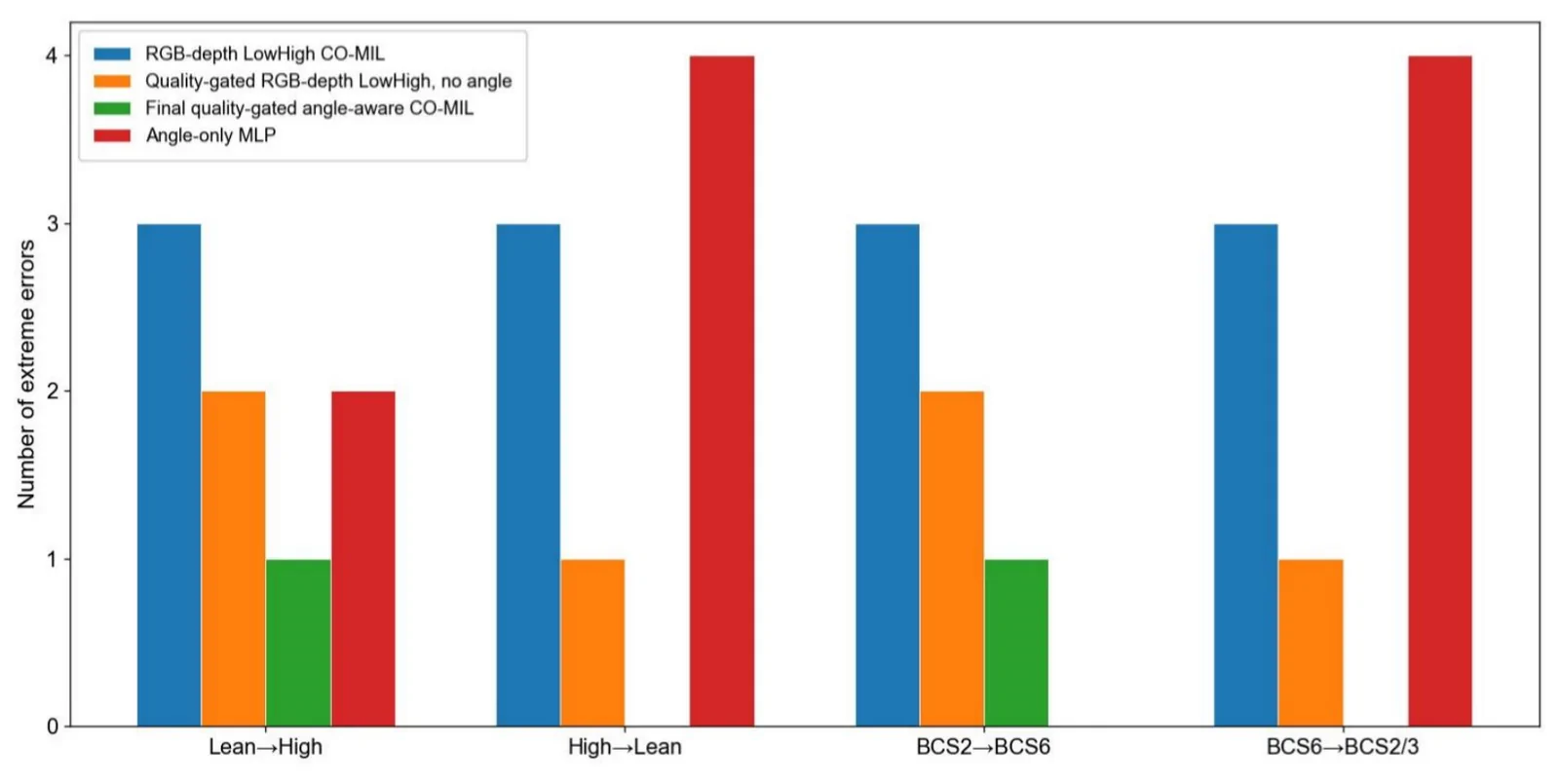
Extreme error comparison between models.

## Data Availability

The original RGB-D dataset used in this study is publicly available in the Dryad repository at https://doi.org/10.5061/dryad.tqjq2bw4s (accessed on 16 September 2026). The source code, processed data indices, model configurations, and analysis scripts supporting the findings of this study are available in the GitHub repository at https://github.com/sxaucao/Episode-Level_Beef_Cattle_Body_Condition_Scoring_with_RGB-D (accessed on 16 September 2026).

## Abbreviations

The following abbreviations are used in this manuscript:

Acc	Accuracy
Attention-MIL	Attention-based multiple instance learning
BCS	Body condition score
CNN	Convolutional neural network
Coverage	Prediction coverage
CO-MIL	Collaborative multiple-instance learning
F1	F1 score
FDR	False discovery rate
MAD	Median absolute deviation
MAE	Mean absolute error
MIL	Multiple instance learning
MLP	Multilayer perceptron
QC	Quality control
QWK	Quadratic weighted kappa
ReLU	Rectified linear unit
RGB	Red, green, and blue
RGB-D	Red, green, and blue-depth
ROS	Robot Operating System
SD	Standard deviation

## Appendix A. Extreme Misclassification Analysis

To evaluate high-risk misclassifications that could lead to opposite assessments of body condition and inappropriate management decisions, four types of extreme errors were further examined: Lean predicted as High, High predicted as Lean, BCS 2 predicted as BCS 6, and BCS 6 predicted as BCS 2 or 3. As shown in Figure A1, the RGB-depth LowHigh CO-MIL model produced three errors in each of the four categories on its own evaluation set. After geometry-based quality gating was introduced without incorporating angle-related features, the corresponding error counts decreased to 2, 1, 2, and 1, respectively. After further incorporating the episode-level dorsal-ridge angle and its reliability-related features, the error counts were reduced to 1, 0, 1, and 0, respectively. In comparison, the Angle-only MLP produced 2 Lean-to-High errors, 4 High-to-Lean errors, no BCS 2-to-BCS 6 errors, and 4 BCS 6-to-BCS 2/3 errors. These results indicate that relying solely on the episode-level median dorsal-ridge angle, the Good-frame ratio, and the transformed Good-angle-frame count is insufficient to reliably distinguish among different body condition states and is particularly prone to severe misclassifications from high to low body condition. Among the current test episodes that satisfied the quality-gating criteria, the final model produced no High-to-Lean misclassifications and no cases in which BCS 6 was misclassified as BCS 2 or 3. However, one Lean episode was misclassified as High, and one BCS 2 episode was misclassified as BCS 6. Overall, the number of extreme errors showed a decreasing trend following the sequential incorporation of geometric quality gating and angle-related features, suggesting that input quality control and geometric information fusion may contribute to reducing high-risk misclassifications involving opposite-direction prediction errors.
